# Comparative Clinical Effectiveness of Nonsurgical Treatment Methods in Patients With Lumbar Spinal Stenosis: A Randomized Clinical Trial

**DOI:** 10.1001/jamanetworkopen.2018.6828

**Published:** 2019-01-04

**Authors:** Michael J. Schneider, Carlo Ammendolia, Donald R. Murphy, Ronald M. Glick, Elizabeth Hile, Dana L. Tudorascu, Sally C. Morton, Clair Smith, Charity G. Patterson, Sara R. Piva

**Affiliations:** 1 Department of Physical Therapy, University of Pittsburgh, Pittsburgh, Pennsylvania; 2 Clinical and Translational Science Institute, University of Pittsburgh, Pittsburgh, Pennsylvania; 3 Institute of Health Policy, Management and Evaluation, Faculty of Medicine, University of Toronto, Toronto, Ontario, Canada; 4 Department of Family Medicine, Alpert Medical School of Brown University, Providence, Rhode Island; 5 Department of Psychiatry, University of Pittsburgh, Pittsburgh, Pennsylvania; 6 Department of Physical Medicine Rehabilitation, University of Pittsburgh, Pittsburgh, Pennsylvania; 7 College of Allied Health, University of Oklahoma Health Sciences Center, Oklahoma City; 8 Department of Medicine, University of Pittsburgh, Pittsburgh, Pennsylvania; 9 Department of Statistics, College of Science, Virginia Polytechnic Institute and State University, Blacksburg; 10 Department of Orthopedic Surgery, University of Pittsburgh, Pittsburgh, Pennsylvania

## Abstract

**Importance:**

Lumbar spinal stenosis (LSS) is the most common reason for spine surgery in older US adults. There is an evidence gap about nonsurgical LSS treatment options.

**Objective:**

To explore the comparative clinical effectiveness of 3 nonsurgical interventions for patients with LSS.

**Design, Setting, and Participants:**

Three-arm randomized clinical trial of 3 years’ duration (November 2013 to June 2016). Analysis began in August 2016. All interventions were delivered during 6 weeks with follow-up at 2 months and 6 months at an outpatient research clinic. Patients older than 60 years with LSS were recruited from the general public. Eligibility required anatomical evidence of central canal and/or lateral recess stenosis (magnetic resonance imaging/computed tomography) and clinical symptoms associated with LSS (neurogenic claudication; less symptoms with flexion). Analysis was intention to treat.

**Interventions:**

Medical care, group exercise, and manual therapy/individualized exercise. Medical care consisted of medications and/or epidural injections provided by a physiatrist. Group exercise classes were supervised by fitness instructors in senior community centers. Manual therapy/individualized exercise consisted of spinal mobilization, stretches, and strength training provided by chiropractors and physical therapists.

**Main Outcomes and Measures:**

Primary outcomes were between-group differences at 2 months in self-reported symptoms and physical function measured by the Swiss Spinal Stenosis questionnaire (score range, 12-55) and a measure of walking capacity using the self-paced walking test (meters walked for 0 to 30 minutes).

**Results:**

A total of 259 participants (mean [SD] age, 72.4 [7.8] years; 137 women [52.9%]) were allocated to medical care (88 [34.0%]), group exercise (84 [32.4%]), or manual therapy/individualized exercise (87 [33.6%]). Adjusted between-group analyses at 2 months showed manual therapy/individualized exercise had greater improvement of symptoms and physical function compared with medical care (−2.0; 95% CI, −3.6 to −0.4) or group exercise (−2.4; 95% CI, −4.1 to −0.8). Manual therapy/individualized exercise had a greater proportion of responders (≥30% improvement) in symptoms and physical function (20%) and walking capacity (65.3%) at 2 months compared with medical care (7.6% and 48.7%, respectively) or group exercise (3.0% and 46.2%, respectively). At 6 months, there were no between-group differences in mean outcome scores or responder rates.

**Conclusions and Relevance:**

A combination of manual therapy/individualized exercise provides greater short-term improvement in symptoms and physical function and walking capacity than medical care or group exercises, although all 3 interventions were associated with improvements in long-term walking capacity.

**Trial Registration:**

ClinicalTrials.gov Identifier: NCT01943435

## Key Points

**Question:**

What is the comparative effectiveness of 3 types of nonsurgical treatment options for patients with lumbar spinal stenosis (LSS)?

**Findings:**

In a randomized clinical trial of 259 patients with LSS, all groups (medical care, group exercise, and manual therapy/individualized exercise) showed improvement in self-reported pain/function and walking capacity at 2 months and 6 months. The manual therapy group had a greater proportion of responders at 2 months, but there were no between-group differences in responder rates at 6 months.

**Meaning:**

Although LSS is a chronic degenerative condition, patients with LSS can show improvement in walking capacity with nonsurgical approaches.

## Teaser

This randomized clinical trial compares the effectiveness of medical care, group exercise, and manual therapy/individualized exercise in patients with lumbar spinal stenosis (LSS).

## Introduction

Lumbar spinal stenosis (LSS) is a degenerative condition of the spine prevalent in 30% of older adults.^1^ Lumbar spinal stenosis is associated with substantial functional limitation of walking, disability, and increased risk of falling.^2,3,4^ Lumbar spinal stenosis accounts for the fastest growth in lumbar surgery in older adults in the United States. The rate of complex fusion procedures for this condition has increased by 137% between 1998 and 2008.^5,6^ These surgical procedures lead to significant costs, risks, complications, and rehospitalizations.^7^ However, evidence is lacking for the effectiveness of nonsurgical interventions and treatment options for patients with LSS.

The North American Spine Society has published a clinical guideline for the treatment of LSS.^8^ The only 2 interventions recommended as evidence based and effective were epidural steroid injection and surgical decompression. This guideline concluded that there was insufficient evidence to make a recommendation for or against the use of nonsurgical treatments, including pharmacologic treatments, physical therapy, exercise, and spinal manipulation. Yet, the only nonsurgical intervention favorably recommended by the North American Spine Society has been contradicted by recent reviews that concluded that the evidence for the effectiveness of epidural injections is of low quality.^9,10^

Since publication of the North American Spine Society guideline, several systematic reviews have corroborated the evidence gap about nonsurgical treatments for LSS.^11,12,13,14,15,16^ To help bridge this gap, we performed a randomized clinical trial to compare the effectiveness of 3 nonsurgical interventions on symptoms and physical function (primary aims) as well as physical activity (secondary aim) in patients with LSS. Exploratory aims included analyses of the number of adverse events, attrition/adherence rates, fall rates, and number of cointerventions.

## Methods

### Study Design

This was a 3-arm, single-center randomized clinical trial. After eligibility confirmation and baseline assessment, patients were assigned using an adaptive allocation with randomization to (1) medical care, (2) group-based exercise, or (3) manual therapy/individualized exercise. All interventions were completed during 6 weeks. Effectiveness and safety outcomes were assessed during 2 follow-up research examinations at 2 months (2 weeks after end of care) and at 6 months after enrollment (4 months after end of care). Data were collected at the Physical Therapy Clinical and Translational Research Center at the University of Pittsburgh.

The study was approved by the University of Pittsburgh institutional review board. All participants were required to provide written informed consent prior to randomization. The trial protocol was previously published^17^ and is available in Supplement 1. This study followed the Consolidated Standards of Reporting Trials (CONSORT) reporting guideline.

### Patient Population

**Figure 1.**
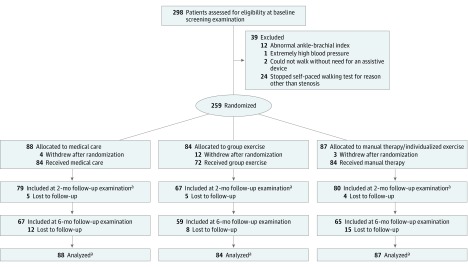
Enrollment of Participants and Study Flow ^a^Primary analysis was comparison of outcome measures from baseline to 2 months. To follow the intention-to-treat principle as closely as possible, data from all participants who were randomized (including dropouts with baseline data but missing follow-up data) were included in the analysis using linear mixed models.

Research participants were recruited from the general population of older adults in the Pittsburgh metro area from November 2013 through June 2016, and analysis began in August 2016. Several recruitment strategies were used, including postcard mailings, research registry, bus advertisements, health fairs, and advertisements in the *Pittsburgh Senior News*. Figure 1 provides a visual summary of the baseline screening, enrollment of participants, and study flow.

Eligible participants were required to have been previously diagnosed as having LSS and to supply magnetic resonance imaging or computed tomography evidence of narrowing of the central canal, lateral recess, and/or foramen. We also confirmed the presence of at least 1 of these clinical signs of LSS: (1) leg symptoms worsened by walking and relieved by sitting; (2) symptoms worsened by lumbar extension and relieved by flexion; and/or (3) leg pain relieved by leaning forward on a shopping cart while walking. Additional inclusion criteria were 60 years or older, ability to read/write English, ability to walk at least 15 meters without an assistive device, limitation of walking due to LSS, ability to engage in mild exercise, and willingness to be randomized. Exclusion criteria were previous surgery for LSS or lumbar fusion, cauda equina symptoms, inability to complete a self-paced walking test (SPWT) for any reason other than symptoms related to LSS, told by a physician not to engage in physical exercise, history of metastatic cancer, severe peripheral artery disease or an ankle-brachial index of less than 0.8, or any neurologic disease other than LSS that affected the ability to walk.

### Randomization

Allocation for each eligible participant occurred immediately after the baseline assessment with the use of a web-based system to ensure concealment of subsequent treatment assignments. The adaptive randomization methodology used a combination of a rank-based method^18^ and a biased coin approach^19^ to balance on 3 continuous baseline prognostic factors: Swiss Spinal Stenosis (SSS) score, SPWT, and age. The adaptive randomization algorithm was created using a structured query language stored procedure written by the systems analyst who developed the electronic data capture system for the study. A detailed description of the adaptive randomization methodology is available in the eAppendix in Supplement 2.

### Treatment Arms

Medical care consisted of 3 visits to a physical medicine physician (R.M.G.) over 6 weeks and primarily involved the prescription of oral medications (first-line treatment), including any 1 or combination of (1) nonnarcotic analgesics (ie, acetaminophen, ibuprofen, celecoxib, or diclofenac); (2) anticonvulsants (ie, gabapentin or pregabalin); and (3) antidepressant agents (ie, nortriptyline, duloxetine, sertraline, trazodone, or mirtazapine). The physician also had the option of referring participants for epidural steroid injections. Indications for injections (second-line treatment) included inadequate pain relief with oral medications, severe neurogenic claudication, and/or patient preference. The physician gave general guidance on gentle stretching and advice to stay active. At each visit, the physician reviewed the response to previous treatment and used shared decision making with each participant to tailor their oral medications and to guide the optional use of epidural injections. This decision making occurred by the physician having comprehensive discussions with participants at each visit regarding their clinical response to medications and offering them the options of staying with the same medication(s), changing to a different type of medication, or having an epidural injection.

The group exercise arm involved participation in supervised exercise classes for older adults at 2 local senior community centers. Participants were asked to attend 2 exercise classes per week for 6 weeks for a total of 12 exercise classes. Each class was about 45 minutes in length and taught by certified senior fitness instructors. Participants self-selected the level of exercise class based on their fitness level, which ranged from easy to medium intensity. Attendance at each class was documented by the community center, then sent to the research team.

The manual therapy/individualized exercise arm involved treatment provided by either a chiropractor or physical therapist. Two chiropractors and 2 physical therapists were trained in the same treatment protocol, with each participant randomly assigned to 1 of these 4 health care professionals. We used clinicians from both professions to increase the clinical generalizability of the treatment protocol because chiropractors and physical therapists can provide manual therapy and individualized exercise instruction. Participants were treated 2 times per week for 6 weeks, with each treatment session lasting about 45 minutes. The clinicians followed a pragmatic treatment protocol that consisted of 3 basic interventions: (1) warm-up procedure using a stationary bicycle; (2) manual therapy procedures, which included lumbar distraction mobilization, hip joint mobilization, side posture lumbar/sacroiliac joint mobilization, and neural mobilization; and (3) individualized instruction in spinal stabilization exercises and home stretching. Each study participant was assessed for specific muscles that required stretching and/or strengthening. The health care professional then developed an individualized program of stretching/strengthening exercises for each patient.

### Outcome Assessments

The primary outcomes were symptoms and physical function measured by a patient-reported outcome (SSS questionnaire^20^) and a performance-based outcome (SPWT^21^). The 12-item version of the SSS form was used, which has a 7-item symptom severity subscale and a 5-item physical function subscale. The range of the SSS score is from 12 to 55 points, with higher scores indicating higher levels of self-reported disability. To our knowledge, there is only 1 study of nonsurgical treatment for LSS that reported a minimal clinically important difference for the 12-item SSS score, which is 3.02 points.^22^ The SPWT is a validated measure of walking performance in patients with LSS that involves participants walking on a level surface until their level of LSS symptoms requires them to stop and sit down to rest. Measurements are recorded for the distance and time walked (30-minute maximum). There is no published minimal clinically important difference for the SPWT used with patients with LSS, to our knowledge.

The secondary outcome was an objective measure of daily physical activity using an armband accelerometer (SenseWear; BodyMedia Inc). The accelerometer was worn 24 hours per day for 7 days, with the expectation to capture at least 4 days with 10 hours of continuous physical activity for data analysis. Physical activity less than 1.5 metabolic equivalent of task units is considered sedentary. We chose the mean number of daily minutes spent in physical activity greater than 1.5 metabolic equivalent of task units as our measure of nonsedentary activity.

We also tracked rates of attrition and adherence to assigned treatment, adverse events, self-reported falls, and cointerventions (exploratory outcomes). We predefined the 2-month follow-up as the primary end point for data analysis. All outcome measures were completed by patients and testers directly on tablet computers, encrypted, and transferred wirelessly to a secure central database.

Blinding of the treating clinicians and research participants was not possible, as both were aware of the intervention they were giving/receiving. We minimized bias by having an independent physical therapist perform all the baseline physical examinations and follow-up reassessments. Our primary outcome measure was a patient self-report questionnaire, and the measure of walking performance was conducted in a manner to minimize examiner bias. Participants were instructed to walk as far as they could until they needed to sit down and rest. The physical therapist only recorded the time and distance walked and was not permitted to coach or provide any encouragement.

### Statistical Analysis

Sample size estimation was based on the primary outcome measure, the SSS questionnaire. At the time of our grant submission, there was no minimal clinically important difference reported in the literature that was derived from a nonsurgical population of patients with LSS. We chose to power the study on a medium effect size of 0.6, resulting in a sample size of 60 participants per group with 80% power (α = .05) to detect a difference between any 2 groups as small as 3.6 points (SD, 6.1) on the total 12-item SSS score with a 20% attrition rate. Subsequently, a minimal clinically important difference of 3.02 points was reported for the 12-item SSS in a nonsurgical population of patients with LSS.^22^ At the request of the funding agency (Patient-Centered Outcomes Research Institute) we continued recruitment for an additional 6 months, resulting in a total of 259 enrolled participants. This resulted in a final power of 83% to detect a 3.02-point difference in SSS scores assuming 20% attrition. No interim analyses were performed; only a final analysis after the end of the extended period of recruitment was performed.

The outcomes and all baseline characteristics were summarized with descriptive statistics, separated by treatment group. Linear mixed models were used to test the differences over time among groups while adjusting for the 3 baseline randomization balancing variables (SSS, SPWT, and age) and repeated measures per participant. We were specifically interested in contrasts at 2 months as the primary time point for analysis. To follow the intention-to-treat principle as closely as possible, data from all participants who were randomized (including dropouts with missing follow-up data) were included in the model. Linear mixed models use all available data for each participant. The normality assumptions were verified for all outcomes.

We also performed a series of secondary responder analyses using dichotomous outcomes, consistent with the recommendations published by the Initiative on Methods, Measurement and Pain Assessment in Clinical Trials,^23,24^ which defines treatment responders as those who achieve at least a 30% improvement relative to each participant’s baseline, which is considered moderate improvement. In this study, we individually dichotomized all participants from each intervention arm into responders or nonresponders based on the criterion of a minimum of 30% improvement between their baseline and follow-up scores for each of the 3 predefined outcome measures: SSS, SPWT, and physical activity. Differences in the proportions of responders between groups were assessed using logistic regression models, controlling for the same randomization variables as those used in the linear mixed models. The α level of statistical significance was set at .05 for both the linear mixed models and logistic regression models. All *P *values were 2 tailed. The linear mixed models used the *F *test for significance, and either χ^2^ or *z *tests were used for the logistic regression models. Analyses were done with SAS version 9.4 (SAS Institute Inc).

## Results

### Patient Characteristics

A total of 259 participants were allocated to medical care (88 [34.0%]), group exercise (84 [32.4%]), or manual therapy/individualized exercise (87 [33.6%]). The groups were relatively balanced on all characteristics except sex and knee osteoarthritis (Table 1). The mean (SD) age was 72.4 (7.8) years, ranging from 60 to 94 years with a mean (SD) body mass index (calculated as weight in kilograms divided by height in meters squared) of 31.0 (6.6). Of 259 participants, 56 (21.6%) were black, 122 (47.1%) did not have a college degree, and 133 (51.4%) had an annual income less than $40 000. Participants had a mean (SD) of 4.7 (2.2) medical comorbidities, including hip (43 [16.6%]) and knee osteoarthritis (82 [31.7%]). At baseline, the mean (SD) SSS score was 31.5 (6.0), and participants walked a median (interquartile range) of 272.7 (130.88-576.40) meters during the SPWT. These levels of baseline values are suggestive of patients with a moderate level of symptomatic LSS.

**Table 1.** Baseline Characteristics of Participants

Characteristic	No. (%)		
	Medical Care (n = 88)	Group Exercise (n = 84)	Manual Therapy/Individualized Exercise (n = 87)
Age, mean (SD), y	72.0 (7.4)	72.9 (8.1)	72.1 (8.1)
Male	42 (48)	45 (54)	35 (40)
Race			
White	68 (77)	66 (79)	67 (77)
Black	19 (22)	18 (21)	19 (22)
Other	1 (1)	0	1 (1)
Married	40 (45)	43 (51)	42 (48)
Household income >$40 000/y	41 (48)	39 (46)	38 (44)
BMI, mean (SD)	31.2 (6.3)	30.8 (6.5)	31.2 (7.1)
Smoking status			
Never	37 (42)	35 (42)	41 (47)
Used to, but quit	41 (47)	46 (55)	40 (46)
Current	7 (8)	2 (2)	6 (7)
Duration of back symptoms, mo			
≤6	7 (8)	12 (14)	7 (8)
>6	81 (92)	72 (86)	80 (92)
Duration of leg symptoms, mo			
≤6	22 (25)	26 (31)	20 (23)
>6	66 (75)	58 (69)	67 (77)
Diagnostic imaging results^a^			
Central canal stenosis	45 (51)	51 (61)	48 (55)
Lateral recess stenosis	66 (75)	65 (77)	69 (79)
Foraminal stenosis	72 (82)	66 (79)	75 (86)
Spondylolisthesis present	48 (55)	45 (54)	56 (64)
Osteoarthritis			
Hip	14 (16)	14 (17)	15 (17)
Knee	32 (36)	21 (25)	29 (33)
No. of comorbidities, mean (SD)	4.9 (2.2)	4.4 (2.2)	4.7 (2.1)
Ankle-brachial index, mean (SD)^b^	1.1 (0.2)	1.0 (0.1)	1.0 (0.2)
Swiss Spinal Stenosis questionnaire, mean (SD)^c^			
Symptom severity subscore	20.1 (4.4)	20.4 (4.2)	20.5 (4.4)
Physical function subscore	11.3 (2.5)	11.2 (2.6)	11.2 (2.5)
Total score	31.3 (5.8)	31.6 (6.0)	31.6 (6.1)
Oswestry Disability Index, mean (SD)^d^	38.1 (11.9)	38.7 (13.5)	38.1 (13.2)
Pain intensity, mean (SD)			
Leg	5.2 (3.4)	5.1 (2.8)	4.9 (2.9)
Back	6.8 (2.6)	6.2 (2.4)	6.5 (2.7)
Gait speed, mean (SD), meters/s	0.9 (0.2)	1.0 (0.2)	0.9 (0.2)
Self-paced walking test, m walked^e^			
Mean (SD)	482.2 (529.1)	433.4 (421.2)	449.2 (485.2)
Median (IQR)	262.9 (119.9-633.7)	286.4 (146.7-556.3)	300.5 (112.7-563.4)
Physical activity, mean (SD), min/d in activities >1.5 MET units^f^	167.4 (130.1)	157.0 (125.5)	172.0 (133.4)

Abbreviations: BMI, body mass index (calculated as weight in kilograms divided by height in meters squared); IQR, interquartile range; MET, metabolic equivalent of task.

^a^ Percentages do not add up to 100 because participants could have more than 1 diagnostic imaging result.

^b^ Normal range is 0.9 to 1.3; lower ratio indicates worse peripheral circulation.

^c^ Symptom severity range is 7 to 35; physical function range, 5 to 20; total score range, 12 to 55; higher scores indicate worse symptoms/function.

^d^ Score range is 0 to 100; higher scores indicate worse function.

^e^ No defined range; total distance walked in 0 to 30 minutes.

^f^ Greater than 1.5 MET units is considered nonsedentary activity.

### Prespecified Outcome Measures

All groups showed some level of improvement on all outcome measures at 2- and 6-month follow-ups (Table 2). Adjusted between-group analyses at 2 months showed that manual therapy/individualized exercise had greater reduction in SSS score compared with medical care (−2.0; 95% CI, −3.6 to −0.4) or group exercise (−2.4; 95% CI, −4.1 to −0.8), but these SSS changes did not reach the level of a minimal clinically important difference of 3.02 points. The mean (SD) baseline total SSS score of participants was about 31 (6.0) points (range, 31.3-31.6; Table 2). Therefore, the magnitude of the differences in reduction of SSS scores between groups (−2.0 vs −2.4 points) represents less than a 10% improvement from baseline and is not likely to be clinically important. Group exercise had greater improvement in mean daily physical activity compared with medical care (28.7; 95% CI, 2.7-54.7). There were no between-group differences found on any outcome measure at 6 months. However, all groups showed within-group improvements in walking distance at 2 months, which was sustained up to 6 months. The mean (SD) walking distance at baseline ranged from 433.4 (421.2) meters to 482.2 (529.1) meters and had increased to a range of 683.3 meters to 723.5 meters at 6 months. The mean within-group improvement in walking capacity ranged from 42% to 67%, a magnitude of change from baseline that could be considered clinically important.

**Table 2.** Analyses of Primary and Secondary Outcome Measures

Time	Outcome Measures							Adjusted Differences, Mean (95% CI)^a^		
	MC		GE		MTE		*P* Value^b^	GE vs MC	MTE vs MC	MTE vs GE
	No.	Mean (SD)	No.	Mean (SD)	No.	Mean (SD)				
**Primary Outcome**										
Swiss Spinal Stenosis questionnaire^c^^,^^d^										
Baseline	88	31.3 (5.8)	84	31.6 (6.0)	87	31.6 (6.1)	NA	NA	NA	NA
2 mo	79	29.1 (6.9)	66	29.8 (5.7)	80	27.2 (5.9)	.01	0.4 (−1.3 to 2.1)	−2.0 (−3.6 to −0.4)^e^	−2.4 (−4.1 to −0.8)^e^
6 mo	67	29.3 (6.8)	59	29.4 (6.7)	65	28.4 (6.7)	.46	−0.5 (−2.3 to 1.3)	−1.1 (−2.8 to 0.6)	−0.6 (−2.4 to 1.2)
Self-paced walking test^d^^,^^f^										
Baseline	88	482.2 (529.1)	84	433.4 (421.2)	87	433.4 (421.2)	NA	NA	NA	NA
2 mo	76	616.6 (620.8)	65	651.5 (639.7)	75	698.6 (662.7)	.26	79.9 (−74.5 to 234.3)	122.9 (−25.7 to 271.6)	43.0 (−111.8 to 197.9)
6 mo	66	683.3 (723.3)	59	688.3 (680.3)	65	723.5 (781.5)	.52	86.5 (−75.7 to 248.8)	73.8 (−84.1 to 231.7)	−12.7 (−175.6 to 150.1)
**Secondary Outcome**										
Physical activity^d^^,^^g^										
Baseline	86	167.4 (130.1)	76	157.0 (125.5)	84	172.0 (133.4)	NA	NA	NA	NA
2 mo	76	148.0 (116.8)	65	170.1 (142.5)	76	176.1 (135.1)	.08	28.7 (2.7 to 54.7)^e^	20.4 (−4.5 to 45.3)	− 8.3 (−34.2 to 17.6)
6 mo	61	159.1 (128.3)	54	155.3 (113.1)	60	161.9 (129.7)	.19	21.3 (−6.9 to 49.4)	−2.9 (−30.1 to 24.3)	−24.2 (−52.5 to 4.0)

Abbreviations: GE, group exercise; MC, medical care; MTE, manual therapy/individualized exercise; NA, not applicable.

^a^ The between-groups linear mixed models were adjusted for baseline Swiss Spinal Stenosis score, self-paced walking test, and age.

^b^ *P* value for omnibus *F* test from linear mixed models for 3-way group comparison at a specified time.

^c^ Total score range, 12 to 55; higher scores indicate worse symptoms and function. The questionnaire included self-reported symptoms and physical function (group × time interaction *P *=* *.03).

^d^ *P* value for omnibus *F* test from linear mixed models for any group × time interaction (at either 2 months or 6 months).

^e^ *P* < .05.

^f^ No defined range; total distance walked in 0 to 30 minutes. Less walking capacity indicates worse physical function. The test included performance-based measure (group × time interaction *P *=* *.52).

^g^ No defined range; greater than 1.5 metabolic equivalent of task units is considered nonsedentary activity. Physical activity-mean daily minutes, >1.5 metabolic equivalent of task units) (group × time interaction *P *=* *.11).

**Figure 2.**
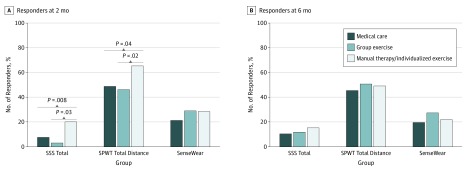
Responder Analyses (≥30% Improvement From Baseline) by Group and Time Logistic regression models were used to compare the between-group proportions, controlling for baseline SSS, SPWT, and age. Only the 4 significant 2-way contrasts at 2 months are depicted by the gray lines and *P *values. All other contrasts at 2 months and 6 months were not significant. SenseWear indicates physical activity; SPWT, self-paced walking test; SSS, Swiss Spinal Stenosis questionnaire.

For analyses using the 30% or more responder criterion, at 2 months, manual therapy/individualized exercise showed greater percentage of SSS responders (16 of 80 [20%]; difference in percentage for manual therapy/individualized exercise vs group exercise [95% CI], −17% [−27% to −7%]; difference in percentage for manual therapy/individualized exercise vs medical care [95% CI], −12% [−23% to −2%]; omnibus *P* = .002) and SPWT responders (49 of 75 [65.3%]; difference in percentage for manual therapy/individualized exercise vs group exercise [95% CI], −19% [−35% to −3%]; difference in percentage for manual therapy/individualized exercise vs medical care [95% CI], −17% [−32% to −1%]; omnibus *P* = .04) compared with group exercise (2 of 66 [3%] and 30 of 65 [46.2%], respectively) or medical care (6 of 79 [7.6%] and 37 of 76 [48.7%], respectively). No between-group differences in physical activity (secondary outcome) responder rates were found at 2 months (difference in percentage for manual therapy/individualized exercise vs medical care [95% CI], −7% [−21% to 7%]; difference in percentage for manual therapy/individualized exercise vs group exercise [95% CI], 1% [−15% to 16%]; difference in percentage for medical care vs group exercise [95% CI], −8% [−22% to 7%]; omnibus *P* = .51), and there were no differences found at 6 months for any of the 3 outcomes (Figure 2).

### Adverse Events, Falls, and Cointerventions (Exploratory Outcomes)

There were greater rates of transient muscle (43 of 80 [54%]; pairwise difference in percentage [95% CI], −22% [−38% to −7%]; *P* < .001) and joint (39 of 80 [49%]; pairwise difference in percentage [95% CI], −32% [−46% to −18%]; *P* < .001) soreness associated with manual therapy/individualized exercise compared with group exercise (21 of 67 [31%]) and medical care (5 of 79 [6%]). Greater rates of gastrointestinal complaints (5 of 79 [6%]; pairwise difference in percentage for medical care vs manual therapy/individualized exercise [95% CI], 5% [1% to 11%]; pairwise difference in percentage for group exercise vs manual therapy/individualized exercise [95% CI], −1% [−4% to 1%]; pairwise difference in percentage for medical care vs group exercise [95% CI], 6% [1% to 12%]; *P* = .06), drowsiness (5 of 79 [6%]; pairwise difference in percentage for medical care vs manual therapy/individualized exercise [95% CI], 6% [1% to 11%]; pairwise difference in percentage for group exercise vs manual therapy/individualized exercise [95% CI], 0% [0%]; pairwise difference in percentage for medical care vs group exercise [95% CI], 6% [1% to 11%]; *P* = .06), and dry mouth (4 of 79 [5%]; pairwise difference in percentage for medical care vs manual therapy/individualized exercise [95% CI], 5% [0% to 9%]; pairwise difference in percentage for group exercise vs manual therapy/individualized exercise [95% CI], 0% [0%]; pairwise difference in percentage for medical care vs group exercise [95% CI], 5% [0% to 9%]; *P* = .12) were reported in the medical care arm compared with the other 2 arms (Table 3). All adverse events were anticipated minor adverse effects that resolved within 48 hours. No serious unanticipated adverse events were found in any group. There were no between-group differences in the number of self-reported falls or medical cointerventions between the end of care and 6-month follow-up. At 6 months, only a small minority of participants reported having spinal surgery (medical care: 2 of 79 [3%]; group exercise: 1 of 67 [2%]; manual therapy/individualized exercise: 1 of 80 [2%]).

**Table 3.** Adverse Events, Falls, and Cointerventions

Variable	No. (%)			*P* Value (χ^2^; 3-Way Omnibus Tests)
	Medical Care	Group Exercise	Manual Therapy/Individualized Exercise	
Minor adverse events at 2 mo (study related but transient/resolved)				
Total No.	79	67	80	NA
Muscle soreness	5 (6)	21 (31)	43 (54)	<.001
Joint soreness	1 (1)	11 (16)	39 (49)	<.001
Gastrointestinal	5 (6)	0	1 (1)	.04
Drowsiness	5 (6)	0	0	.01
Dry mouth	4 (5)	0	0	.04
Headache	4 (5)	0	1 (1)	.11
Serious adverse events at 2 mo (study related and requiring outside medical treatment)	0	0	0	>.99
Falls at 6 mo (between end of care and 6 mo)				
Total No.	66	59	65	NA
0	35 (53)	38 (64)	34 (52)	.32
1	17 (26)	15 (25)	21 (32)	.60
≥2	14 (21)	6 (10)	10 (15)	.25
Cointerventions at 6 mo (between end of care and 6 mo)				
Total No.	67	59	65	
Added use of assistive device	6 (9)	6 (10)	10 (15)	.47
Spinal injections	7 (11)	8 (14)	7 (11)	.84
Added or increased pain medications	8 (12)	11 (19)	7 (11)	.39
Stopped or decreased pain medications	3 (5)	1 (2)	2 (3)	.87
Spinal surgery	2 (3)	1 (2)	1 (2)	>.99

Abbreviation: NA, not applicable.

## Discussion

Current guidelines provide scant information about the safety and/or effectiveness of nonsurgical interventions for LSS. This study provides new evidence about group exercise in community centers and manual therapy/individualized exercise provided by physical therapists and chiropractors. It appears that the chiropractic/physical therapy intervention had better short-term outcomes at 2 months but that none of the interventions were superior to each other at 6 months. However, all groups showed clinically important improvement in their walking distance, which was sustained at 6 months. The medical care group did not receive any specialized exercise instruction, so it is unclear why they showed improvements in their walking capacity. One possible explanation is that all interventions led to a reduction in fear avoidance behavior and/or passive coping, giving patients more self-confidence to try walking further. This has clinical relevance because reduced walking performance is the dominant physical impairment cited by patients with LSS. These results suggest that although LSS is a chronic degenerative condition with waxing/waning of symptoms, not all patients show progressive physical deterioration; some patients can still make improvements in their physical function (walking capacity) without surgery.

There was an intriguing finding relative to the group differences on the primary outcomes. Although participants reported only modest improvement in self-reported symptoms and physical function (using the SSS questionnaire), they had more substantial increases in their walking capacity (using the SPWT). One possible explanation is that the items of the SSS questionnaire may be more responsive in the patients with severe LSS who undergo spine surgery and experience a greater magnitude of change in their symptoms and physical function after surgery. It is also possible that information about walking capacity obtained indirectly from a patient self-report (SSS questionnaire) may not be as accurate as a direct measurement of time and distance walked (using the SPWT).^25^ Both of these possible explanations need to be further explored in future research trials.

This study was designed as a comparative effectiveness trial, looking for the most effective of these 3 interventions. In real clinical practice, it might serve patients best if their health care professional would discuss these therapeutic options within the context of shared decision making. Any of these approaches seem to be a reasonable option for patients with LSS who choose not to undergo surgery. It is also possible that various combinations of these interventions might have a synergistic or sequential effect and provide patients with LSS more clinical benefit than any one individual intervention. However, the study design did not explore the comparative effectiveness of various combinations of these interventions used in a multimodal manner.

### Limitations and Strengths

There were some limitations associated with this study. A greater proportion of participants withdrew from group exercise immediately after randomization. This may have created selection bias as those participants who chose to accept randomization into group exercise may have been more motivated toward physical activity than participants in the other 2 arms. Increased motivation might be a confounding variable in the results showing greater physical activity in this group at 2 months. Also, participants who received manual therapy/individualized exercise spent about 45 minutes face to face with a physical therapist or chiropractor for 12 sessions. This increased personal attention might be a confounding variable in the results showing greater short-term improvement in self-reported pain and function. Also, it is not possible to determine whether the manual therapy or individualized exercise component had an independent treatment effect because the treatment protocol was a combination of these 2 treatment methods.

Another potential limitation is that we cannot rule out the possibility of general improvement due to natural history since we did not include a no-treatment arm. Understanding the natural history of LSS is challenging because most published LSS clinical trials have not included a no-treatment arm, to our knowledge. However, a 2017 observational study of the natural history of 146 nonsurgical patients with LSS followed up over 3.3 years found spontaneous improvements for pain and health-related quality of life but not for walking capacity.^26^ This suggests that the improvements in walking capacity found in the present study were not simply an artifact of natural history.

The demographic and baseline characteristics of the participants were comparable with those enrolled in previously published LSS trials, which adds to the generalizability of our results. Also, the pragmatic nature of the interventions should facilitate the adoption of the study interventions by many stakeholders. Our tailored approach to medical care did not include the use of any opioids and could be easily adopted by primary care physicians. Group exercise classes are routinely available at most community centers at little or no cost to older adults. The manual therapy/individualized exercise protocols could be readily implemented by most chiropractors and physical therapists with relatively minimal training. However, the cost of 12 treatments with these health care professionals is greater than group exercise classes or 3 visits to a physician.

## Conclusions

Mounting concern about the rising rates of spine surgery and opioid use in older adults makes a compelling case for the dissemination of new evidence about safe and effective nonsurgical and nonopioid pharmacologic treatment options for LSS. The results of this study provide new evidence about the comparative effectiveness of tailored medical care, group exercise, and a combination of chiropractic/physical therapy as viable nonsurgical and nonopioid treatment options for patients with LSS. Patients, health care professionals, and other stakeholders would benefit from the dissemination of these new research findings.

## Notes

Supplement 1. **Trial Protocol**

Supplement 2. **eAppendix.** Trial baseline adaptive randomization methodology

Supplement 3. **Data Sharing Statement**
