# Supplementary material for: Comparative Clinical Effectiveness of Nonsurgical Treatment Methods in Patients With Lumbar Spinal Stenosis: A Randomized Clinical Trial
Source: JAMA Netw Open. 2019 Jan 4;2(1):e186828. doi: 10.1001/jamanetworkopen.2018.6828 (PMC6324321; doi:10.1001/jamanetworkopen.2018.6828)
Supplement: Supplement 1. — Trial Protocol [file jamanetwopen-2-e186828-s001.pdf]

Clinical Intervention Study Protocol

A Comparison of Non-Surgical Treatment Methods for Patients with Lumbar Spinal Stenosis

Principal Investigator:

Michael J. Schneider, DC, PhD

Supported by:

Patient Centered Outcomes Research Institute (PCORI)

Version 1.0

April 2013

CONTENTS

1. STUDY OBJECTIVES 4
1.1. Primary Aim 4
1.2. Secondary Aim 4
1.3. Exploratory Aim 1 4
1.4. Exploratory Aim 2 4
2. BACKGROUND 5
3. STUDY DESIGN 6
3.1. Overview of Study Design 6
3.2. Summary of Study Recruitment and Screening Procedures 9
4. SELECTION AND ENROLLMENT OF SUBJECTS 9
4.1. Inclusion/Exclusion Criteria 9
4.2. Telephone Screening Process and In-person Eligibility Screening Visit Scheduling 10
4.3. Baseline Screening and Exam 10
5. BASELINE TESTING 11
5.1. Clinical Examination 11
6. RANDOMIZATION 16
7. MASKING 16
8. STUDY INTERVENTIONS 16
8.1. Usual Medical Care 16
8.2. Community-based Group Exercise 17
8.3. Clinic-Based Manual Therapy and Rehabilitative Exercise 17
9. FOLLOW-UP VISITS 18
10. ADVERSE EVENTS 19
10.1. Management 19
10.2. Report 19
11. STUDY COMPENSATION 19
13. DATA ANALYSIS 20
13.1. Descriptive Statistics 20
13.1. Analytic Statistics 20

14. DROPOUT AND MISSING DATA 22
15. RECORD KEEPING 22
16. DATA MANAGEMENT PLAN 22
17. QUALITY ASSURANCE 23
18. HUMAN SUBJECTS 23
18.1. Institutional Review Board (IRB) Review and Inform Consent 23
18.2. Potential Risks/Risk Management 23
18.3. Privacy 24
18.4. Confidentiality 25
18.5. Potential Benefits of the Proposed Research to Human Subjects and Others 25
18.6. Importance of the Knowledge to be Gained 25
APPENDIX *(documents from appendix are available upon request)* 26
A. Study Forms 26
B. Informed Consent 26
C. Study Advertisement 26

Table of Figures

Figure 1- Study Design 8

Table of Tables

Table 1. Measures completed at each time point 9

1. STUDY OBJECTIVES

This study is a pragmatic comparative effectiveness study of three types of non-surgical treatment options for patients with Lumbar Spinal Stenosis (LSS). The main goal is to provide evidence to inform the choices between three types of non-surgical treatment options for patients with LSS. The three comparison groups in this trial are:

- Group 1: Usual medical care
- Group 2: Community-based group exercise classes
- Group 3: Clinic-based manual therapy and rehabilitative exercise

1.1. Primary Aim

To compare the clinical outcomes between these 3 treatment groups using two validated primary outcome measures of pain and physical function; Swiss Spinal Stenosis questionnaire (self-report measure of pain/function) and Shuttle Walk Test (performance-based measure of function).

- Hypothesis: LSS subjects in Groups 2 and 3 will demonstrate greater improvement in self-reported pain/function and walking performance as compared to Group 1.

1.2. Secondary Aim

To compare changes in physical activity between these 3 treatment groups using the SenseWear Armband (real-time measure).

- Hypothesis: Subjects in Groups 2 and 3 will demonstrate a greater change in physical activity compared to Group 1.

1.3. Exploratory Aim 1

To explore relationships between 6-month attrition rates, number of adverse events, adherence rates, number of falls, and co-interventions across treatment groups.

- Hypothesis: For all 3 treatment groups there will be a low incidence of adverse events and similar treatment adherence rates. The 6-month attrition rate, number of falls, and co-interventions will be lower in Groups 2 and 3 compared to Group 1.

1.4. Exploratory Aim 2

To explore potential baseline predictors of treatment response in each of the 3 treatment groups.

- Hypothesis: A group of baseline physical, psychosocial, and demographic measures will be associated with treatment response and non-response in each group. We expect baseline predictors of treatment response to be different in Group 1 as compared to Groups 2 and 3.

2. BACKGROUND

Lumbar spinal stenosis (LSS) is the most common reason for spinal surgery in older adults. It has been shown that surgery is effective for severe cases of LSS, but many patients with mild to moderate symptoms are not surgical candidates. These patients and their providers are seeking effective non-surgical treatment methods to manage their symptoms; yet there is a serious lack of comparative effectiveness research in this area.

LSS is a degenerative arthritic disease of the spine, which is often associated with significant functional limitations of walking and disability. Radiographic and clinical data from the Framingham cross-sectional study reports the prevalence of degenerative lumbar stenosis at 30%. The hallmark symptom of the clinical syndrome of LSS is significant leg pain. The leg pain is typically worse with walking, which often leads to a dramatic impairment in ambulation and increased risk of falling; comparable to patients with severe knee osteoarthritis. Decreased ambulation leads to decreased physical activity, which can negatively impact overall health. Limitation of physical activity is associated with obesity, heart disease, peripheral artery disease, diabetes, and cognitive decline. Impairment of walking ability can also have a negative impact on the ability of older adults to stay in the workforce and live independently.

The findings of our research may have a strong impact on the efficiency of patient care in several ways. As noted previously, LSS is the most common reason for spinal surgery in older adults, with a lack of clear guidelines about the best choices for non-surgical care options. Patients, providers, payers and policy makers are all left in a quandary about how to make informed decisions about their respective management of LSS. Research evidence is urgently needed to inform all of these stakeholders about the clinical benefits and risks of the common non-surgical approaches being compared in this proposed study.

Patients with LSS need more information about the benefits and risks of the non-surgical treatment options being offered to them. Primary care physicians and surgeons who are caring for LSS patients also report that they simply don't have enough good evidence to inform their choices about which non-surgical treatments to recommend for their patients. This includes the spine surgeons and neurosurgeon who have provided the PI with letters of support for this study. They have indicated being unsure of the best clinical approach to take with LSS patients for whom they have determined surgery is not warranted or contraindicated. This proposed study should help to provide both patients and providers with meaningful information to better guide their treatment choices.

This research will also provide evidence that may influence changes at the level of payers and policy makers. One of the key stakeholders cooperating with this proposed study is the University of Pittsburgh Medical Center (UPMC) Health Plan. The UPMC Health Plan recently instituted a policy mandating a three month course of chiropractic, physical therapy, and oral medication before authorization of any spine surgery for chronic back pain, including spinal stenosis. The senior medical director indicated that internal research at the UPMC Health Plan uncovered the fact that less than 50% of patients undergoing spine surgery had been treated by a physical therapist or chiropractor prior to their surgery. The percentage of lumbar spinal stenosis patients receiving non-surgical treatment prior to surgical intervention was even lower. These low utilization rates are of particular

concern to the Health Plan, because it implies that LSS patients are not accessing many potentially beneficial non-surgical options.

It is also possible that the results of our study will provide enough evidence to stimulate other larger payers and policy makers to follow the lead of the UPMC Health Plan, and provide incentives to patients and providers to explore a trial of non-surgical treatment before considering spine surgery. In the absence of good evidence, patients with LSS and the providers who care for them are left feeling uncertain about the benefits and risks of group exercise classes, manual therapy and rehabilitative exercise for LSS. We believe the results of our study will remove some of this uncertainty and thereby have a strong impact on health care performance at the level of patients, providers, payers and policy makers.

3. STUDY DESIGN

3.1. Overview of Study Design

This study is a prospective randomized controlled clinical trial of 180 older adults who have both an anatomic diagnosis of LSS and signs and symptoms consistent with a clinical diagnosis of LSS confirmed by clinical examination. Eligible subjects will be randomized into one of three pragmatic treatment groups: 1) usual medical care; 2) community based group exercise; or 3) individualized manual therapy and rehabilitative exercise. All subjects will be treated for a 6-week course of care. Follow-up evaluations will occur at 2 and 6 months after randomization. Figure 1 gives an overview of study design.

Figure 1: Diagram of Study Flow

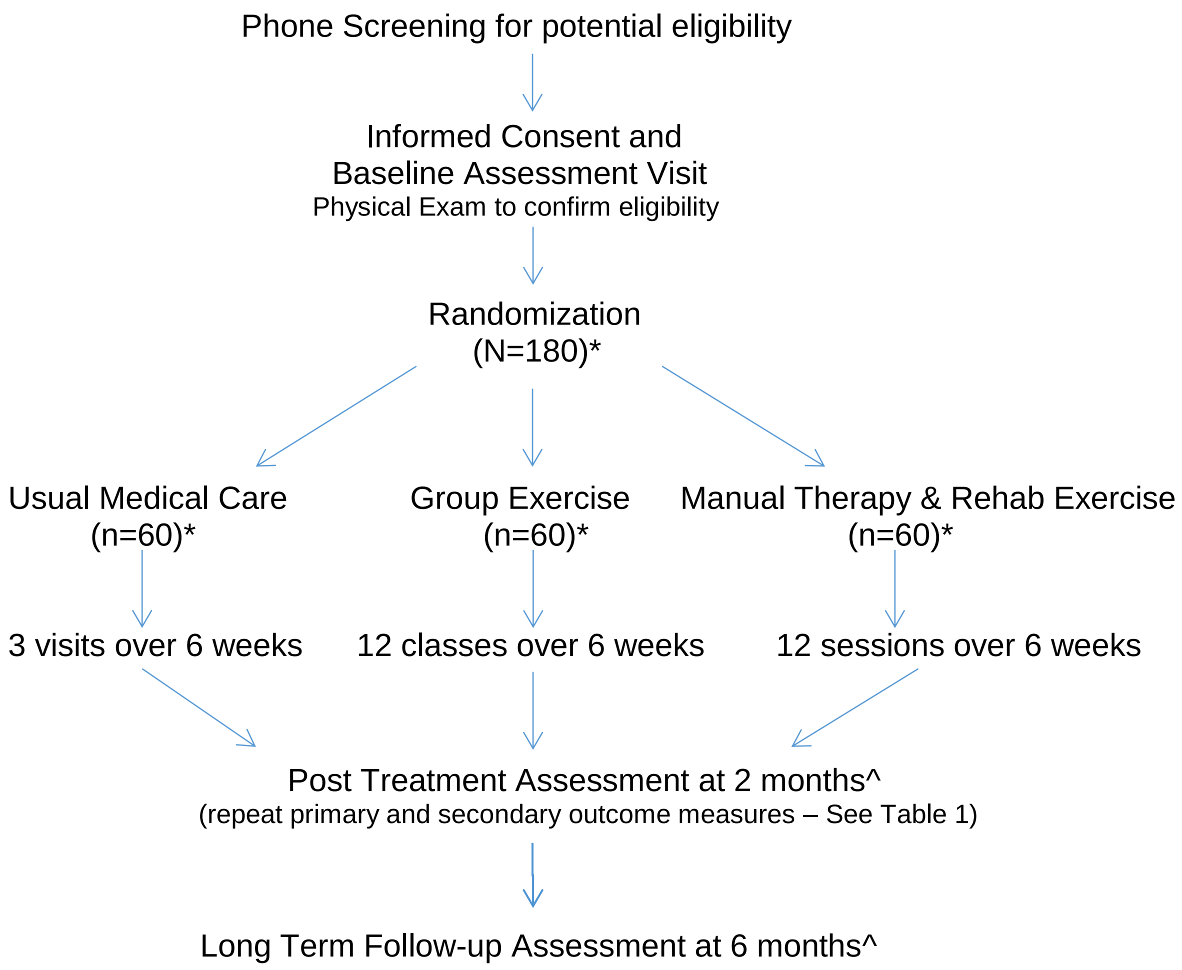

*Sample size of N=180 allows for a 15% attrition rate. Power analysis was based on n=50 per group (N=150)

^Post treatment and long term follow-up assessments will repeat the primary (SSS, SWT) and secondary (SWA) outcome measures

Abbreviation Key
SSS = Swiss Spinal Stenosis questionnaire
SWT = Shuttle Walk Test
SWA = SenseWear Armband

Table 1: Timeline of Outcome Measures				
Measure	Baseline	Mid-Treatment	Follow Up 2 months	Follow Up 6 months
Primary Outcomes: Physical Function				
Self-report=SSS	x	x	x	x
Performance-based=SWT	x		x	x
Secondary Outcomes				
Physical Activity Real-time = SWA*	x		x	x
Falls History	x		x	x
Balance Confidence Scale (ABC)	x		x	x
Oswestry	x	x	x	x
Side Effects, Adverse Events		x	x	x
RAND-36	x		x	x
SPPB	x		x	x
PGIC/Satisfaction			x	x
Other Variables				
Demographics	x			
Co-morbidities (MCDI)	x			
Baseline History/Physical Exam	x			
Psychosocial				
Fear (TSK)	x			
Depression (PROMIS)	x			
Expectancy (CEQ)	x			

Abbreviation Key:
SSS=Swiss Spinal Stenosis questionnaire
SWA = Shuttle Walk Test
SWA=SenseWear Armband
MCDI=Modified Co-morbidity Disease Index
SPPB= Short Physical Performance Battery
ABI=Ankle Brachial Index
TSK=Tampa Scale for Kinesiophobia
PROMIS= Patient Reported Outcomes Measurement Information System
CEQ=Credibility/Expectancy Questionnaire
PGIC= Patient Global Index of Change

3.2. Summary of Study Recruitment and Screening Procedures

The study uses several recruitment strategies with the intent of enrolling 8 subjects each month. The primary strategy comprises of direct mailings of postcards to adults age 60 and above residing in local neighborhoods.

Additional recruitment strategies include physician referrals, study letters sent to participants of research registries (Clinical and Translational Science Institute -CTSI - Registry and the Pittsburgh Claude D. Pepper Older Americans Independence Center Registry), advertisements on local radio stations, newspapers, and other publications. The study also recruits subjects directly from the Vintage Community Senior Center and the Squirrel Hill Jewish Community Center. Both centers are designated community senior citizen centers by the Allegheny County Area Agency on Aging. The directors of these centers have agreed to assist with the study's recruitment efforts by allowing posters and informational brochures to be placed in their facilities, by sending e-blasts to their respective members, and by posting announcements about the research study in their newsletters.

For all recruitment efforts, we use services from the University of Pittsburgh - University Marketing Communications (UMC). The UMC is a resource available to researchers at the University of Pittsburgh, which has a full-time staff that provides advertising, planning, copywriting, design, and production services and handles reservation of newspaper space.

All recruitment materials have the research coordinator's telephone number so that subjects may contact the research team, if interested in the study. Thus, regardless of the recruitment method, the potential subjects initiate contact with study personnel. All individuals who call to inquire about the study give their verbal consent to undergo telephone screening. Those deemed potentially eligible over the phone will be scheduled for an in-person assessment to confirm eligibility.

4. SELECTION AND ENROLLMENT OF SUBJECTS

4.1. Inclusion/Exclusion Criteria

Inclusion Criteria: Subjects will be **included** in the study if they:

- Are older than 60 years of age.

- Have a clinical history of lumbar spinal stenosis (LSS)
- Have imaging (CT or MRI) evidence of LSS
- Have the ability to read/write English and understand instructions
- Have limitation of standing and/or walking tolerance (neurogenic claudication)
- Agree to attend 2 treatments per week for 6 weeks
- Have the ability to engage in mild exercise
- Can walk 100 feet without need for an assistive device
- Are willing to be randomized to one of the 3 treatment groups

Exclusion Criteria: To ensure safety, participants will be **excluded** from the study if they:

- Have a history of metastatic cancer
- Had previous lumbar surgery for LSS; i.e. laminectomy and/or spinal fusion
- Currently have cauda equina symptoms including saddle paresthesia
- Have a history of severe Peripheral Artery Disease and/or Ankle Brachial Index < 0.8 (vascular claudication)
- Have been told by a physician that they should not engage in aerobic exercise
- History of neurologic (e.g., cervical myelopathy, stroke) or neuro-degenerative (e.g., Parkinson's) disease other than LSS that affects the subject's ability to walk
- Cannot complete a walking test for any reason other than symptoms related to LSS (e.g. chest pain, severe hip arthritis, etc.)

4.2. Telephone Screening Process and In-person Eligibility Screening Visit Scheduling

All potential research participants recruited to this study will first be screened by telephone for eligibility criteria by research assistants who will be trained to follow a screening script and checklist. The checklist will include the key inclusion/exclusion criteria that can be evaluated by phone. Potential participants who pass the telephone screen will be scheduled for a screening/baseline appointment at the PT-CTRC for further evaluation.

The research coordinator mails or emails a copy of the informed consent document, driving directions to get to the research facility and a letter with general guidelines for the entire visit.

The appointment is confirmed with the subject one day prior to the scheduled visit. The research coordinator must contact the subject to verify if the subject has any questions about the study (after reading the informed consent), remind subject to bring an updated list of medications, discuss transportation and directions, and re-confirm or reschedule the appointment.

4.3. Baseline Screening and Exam

The purpose of the baseline evaluation is to confirm eligibility and collect baseline data. Personnel performing the procedures: Licensed Physical Therapists or Doctors of Chiropractic, and/or Research Coordinator. Location of the procedures: Physical Therapy Clinical & Translational Research Center (PT-CTRC): a component of the University of Pittsburgh Clinical Translational Science Institute (CTSI) and is a state-of-the-art outpatient facility located in Forbes Tower at the University of Pittsburgh main campus.

Duration of the procedures: The Baseline Screening and Exam will take approximately 2 hours.

At the in-person baseline screening visit, the research coordinator explains the study to the potential subject and clarifies any questions the subject might have. The consent form is reviewed using a powerpoint presentation that explains the key elements. If the subject has no questions or after addressing the subject's concerns, both the coordinator and the subject sign the informed consent (a signed copy is given to the subject and the original is kept in a locked research file).

All subjects are asked to complete a Contact Information Form that is used for compensation purposes (i.e., to assign a pre-paid card to the subject).

The following questionnaires are completed: Demographics, Current Medications

The research coordinator confirms the pre-screening self-reported eligibility/ineligibility criteria.

At any point during the screening process if a subject is deemed ineligible the evaluation is terminated and the subject is compensated for his/her time.

If the subject is deemed eligible for the study, the research coordinator introduces the subject to the tester who continues onto baseline eligibility exam component of this visit.

5. BASELINE TESTING

5.1. Clinical Examination

Baseline symptoms and clinical history These questions are asked verbally while subject is lying supine in preparation for the ABI test:

1. Duration of leg symptoms
2. Duration of back symptoms
3. Predominant pain: legs, low back, equal
4. VAS intensity of leg pain (0-10)
5. VAS intensity of back pain (0-10)
6. Do symptoms improve when bending forward?
7. Do symptoms improve when bending backward?
8. Do symptoms occur when standing up?
9. Is your ability to walk impaired by leg pain?
10. Does your leg pain improve once you sit down and rest?
11. Does leaning forward on an assistive device allow you to walk farther? (shopping cart, cane, walker)
12. Pain extends to: buttock, thigh, calf/foot
13. Do you have urinary incontinence? If yes, does it happen only when walking?
14. Qualitative description of leg symptoms: a) Neurologic: numbness, tingling, pins & needles, burning or b) Pain
15. Have you had hip/groin pain for most days in the past month?

16. Have you had knee pain for most days in the past month?>
17. Do you have morning hip/groin stiffness? < 60 minutes duration?
18. Do you have morning knee stiffness? < 30 minutes duration?
19. Pain present on internal hip rotation? (demonstrate)

2. **Ankle Brachial Index (ABI)**: We will use a standard hand-held Doppler ultrasonic device and sphygmomanometer to record the systolic and diastolic pressures over the dorsal pedis, posterior tibial, and brachial arteries. The ABI is the ratio of the lower to upper extremity systolic pressures, with the normal range being >0.9. We will exclude patients with an ABI <0.8 at the baseline from participation in our study, and will offer to send a report of our findings to their primary care physician for further evaluation

The subject must lie supine with the socks/shoes off for about 10 minutes below performing the ABI. Make sure the feet are not cold. It is okay to cover the feet with light blanket or sheet while they are acclimating to the room temperature. Use the Doppler unit to find the pulse over the medial malleolus (posterior tibial artery) and then pump up the blood pressure cuff until the sound disappears. Slowly release the pressure and record the systolic pressure as the moment the sound re-appears. The diastolic pressure is recorded when the sound disappears again. Repeat on opposite ankle. Then perform brachial blood pressure readings at both elbows. Record all results directly into the iPad. For small framed patients, use the child-sized cuff if necessary over the ankles. If the cuff is too large, it could lead to an inaccurate reading.

Make sure to confirm that the patient is eligible for the study after completing the ABI procedure. If the iPad tells you the patient is ineligible at this point in the exam, please inform the patient that the results of this test indicate that they need to contact their primary care physician about the possibility of PAD.

2. **Height and weight:** record height in centimeters and weight in kilograms.

3. **Neurological Exam**: consists of 5 tests performed bilaterally. The purpose of the neuro exam is to give the patient a complete physical examination that would typically be performed by a physical medicine and rehabilitation physician or spine surgeon. We will not be excluding patients from the study based upon their neuro exam findings. However, we would alert the patient to any of these findings that we feel should be shared with their primary care physician: positive Babinski, severe loss of muscle function, pinprick or vibration sense.

1) Reflexes: knee and ankle - recorded as Absent, Normal or Hyper. Sluggish or slightly exaggerated reflexes will be collapsed into the category “Normal”. Only clearly hyper-reflexic responses will be recorded as “Hyper”

2) Vibration sensation: We will use the bio-thesiometer to quantify vibratory sensation at the great toe and medial malleolus. Patients will be familiarized with the sensation by turning the amplitude to maximum and then tested by gradually increasing the amplitude from zero and asking when they first feel the sensation. The lowest of two readings will be recorded.

3) Pinprick sensation exams will be performed over 3 anatomical regions: medial ankle, dorsal foot, and lateral ankle. These areas correspond respectively to the L4, L5, and S1 dermatomal distributions.

Record three findings: Absent, Diminished or Normal. The finding of hypersensitivity will be collapsed into the category of "normal".

4) The Babinski sign is also known as a positive response to the plantar reflex test, which is used to asses motor neuron function. To test the reflex, the lateral, or outside, sole of the foot is stroked with a thumbnail or blunt instrument. A positive Babinski sign is when the big toe extends and the other toes fan out in an "upgoing" manner, which is considered an abnormal neurological response indicative of upper motor neuron damage.

5) Manual Muscle testing will be performed by having the patient contract the muscle against resistance of the clinician's hands. The standard clinical grading system will be used to rate the degree of muscle strength from a minimum of 0 (complete paralysis) to 5 (normal strength). We will test plantar flexion of the foot and dorsiflexion of the great toe and foot.

4. **Neurodynamic Exam**: this procedure is designed to place tension on the lumbar nerve roots by keeping one end of the neural tract stationary and applying tension to the other end. This is typically performed in seated position with the patient's neck and upper back in a flexed posture, while the clinician passively extends the knee and raises the lower extremity into hip flexion. Additional neural tension can be added by passively dorsiflexing the foot/ankle. A positive test is reproduction of significant leg pain/symptoms, which will be recorded as 2 possible categories of response: Positive, Negative. Two nerves will be tested: Sciatic and Femoral. A positive test is defined as any reproduction of symptoms verbalized by the patient as a "familiar pain" in the leg or buttock region during performance of the test. The report of a feeling of "muscle tightness" will be recorded as a negative test.

5. **Hip and Knee exam**: We will apply the American College of Rheumatology (ACR) criteria for hip osteoarthritis (OA) and knee OA, recording whether or not the patient meets either/both of these criteria.

Hip Exam:

The patient will be lying in the supine position and the therapist will use a goniometer or inclinometer to measure (in degrees) the amount of internal rotation and flexion range of motion in each hip. The therapist will manually input the answer Yes or No on the iPad, for each ROM and each hip; using these criteria:

- Internal rotation: YES = < 15 degrees NO = > 15 degrees
- Flexion: YES = < 115 degrees NO = > 115 degrees

We will apply the American College of Rheumatology (ACR) criteria for hip osteoarthritis (OA) as defined below:

History of hip/groin pain for most days of the prior month, in addition to at least 3 of the following:

1. Pain present on internal hip rotation
2. Morning stiffness <60 minutes duration
3. Age > 50 years
4. Internal hip rotation < 15 degrees
5. Hip flexion < 115 degrees

The computer will automatically combine these physical exam findings with the previous self-report questions, with a pop-up window alerting the clinician whether these ACR criteria have been met for a diagnosis of hip OA.

Knee Exam:

The patient will be lying in the supine position and the therapist will proceed to palpate the medial and lateral joint lines of the knee with one hand, while passively flexing and extending the knee with the other hand. A "yes" response to crepitus is recorded if there is any audible or palpable clicking, grinding, or cracking during this procedure. The therapist will also record (yes or no) whether the palpation revealed any bony enlargement, tenderness, or warmth over the knee structures. We will apply the American College of Rheumatology (ACR) criteria for knee osteoarthritis (OA) as defined below:

Articular knee pain present for most days of the prior month, in addition to at least 3 of the following:

1. Crepitus on active joint motion
2. Morning stiffness <30 minutes duration
3. Age >50 years
4. Bony enlargement of the knee on examination
5. Bony tenderness of the knee on examination
6. No palpable warmth

The computer will automatically combine these physical exam findings with the previous self-report questions, with a pop-up window alerting the clinician whether these ACR criteria have been met for a diagnosis of hip OA.

6. **Lumbar Spine Exam**: this will involve the simple task of having the patient perform single movements of lumbar Active Range of Motion (AROM) in flexion and extension, observing the movements and asking the patient about pain provocation and peripheralization:

1. Was back pain provoked during extension?
2. Was back pain provoked during flexion?
3. Did you feel pain in your leg, calf or foot during extension?
4. Did you feel pain in your leg, calf or foot during flexion?

7. **Extended Short Physical Performance Battery (SPPB)**. We will administer an extended version of the SPPB. The published SPPB protocol is a reliable and valid set of tests that assesses 3 lower extremity performance subscales. We will add the 4th test: 1) Static standing balance; 2) Four meter gait speed; 3) Five repeated chair stands; 4) Ability to stand on one leg.

8. **Shuttle Walk Test**. This is validated performance-based measure of physical function (walking) that has been used in LSS research. The SWT is well tolerated by older adults and is relatively quick; it is completed within a maximum of 12 minutes. No special equipment or large area is required for walking, making it an ideal test for most out-patient clinical settings. The test can easily be performed by any healthcare provider by following the following test protocol.

A 10-meter course (one shuttle) is measured on flat ground without obstacles and is marked. A tape recorder plays the SWT prerecorded tape, which explains the test to the patient. The patient must reach the end of the walkway within a specified time dictated by a beep sounding from the tape. During the first minute of the test, beeps sound each 20 seconds and three shuttles (30 m) are completed. During the second minute, four shuttles are completed; during the third minute five shuttles are completed; and so on up to 14 transits in 12 minutes,

with a maximum total distance of 1020 m. The assessor counts the number of completed shuttles. The test ends when the patient can no longer complete a shuttle before the next beep sounds. If the patient is within 50 cm of the end of the shuttle when the beep sounds, he or she is given the opportunity to make up the distance during the next shuttle. The result of the test is given in meters (number of completed shuttles multiplied by 10).

Participants who meet the eligibility criteria following this baseline appointment will be set up with the SenseWear Armband to wear at home for one week.

9. **The SenseWear Armband** - Real-time physical activity will be measured by the SenseWear Professional v 6.1 (Body Media Inc, Pittsburgh PA). The SenseWear armband (SWA) is a small device that collects information from multiple sensors: a triaxial accelerometer, heat flux, skin temperature, and galvanic signal. The information is integrated and processed by software using proprietary algorithms utilizing subjects' demographic characteristics (gender, age, height, and weight) to provide minute-by-minute estimates of physical activity. The SWA has shown good reliability and validity . Subjects will be instructed on the proper application of the SWA on the upper arm at each assessment visit and given a unit to take home. Subjects will be instructed to wear the SWA on the back of the right arm during 24 hours/7 days (except during shower or water activities) to obtain 5 whole days of data. After 7 days of use, subjects will mail the device back in a pre-paid Priority Mail package.

Self-Reported Measures:

The research assistant provides the instructions and necessary assistance to subjects to complete the self-reported forms.

1. **Swiss Spinal Stenosis Questionnaire (SSS)**: This is our primary outcome measure. The SSS instrument is a patient self-report measure of pain and physical function. This instrument will be repeated at mid-treatment, post treatment, and long term follow up.

2. **Falls History**: patient self-report of falls over the past year (4-9 items).

3. **Modified Co-Morbidity Disease Index (MCDI)**: subjects will be asked to indicate any medical conditions with which they have been formally diagnosed by a physician in the past.

4. **Global Walking and Mobility Items**: self-rating of walking ability and mobility.

5**. Activities Specific Balance Confidence (ABC) Scale**: subject's level of self-confidence about performing certain activities of daily living without losing balance or becoming unsteady.

6. **Oswestry Low Back Pain Disability Index**: self-report measure of interference due to back pain.

7. **The RAND 36-Item Health Survey**: a validated and well-used measure of general Quality of Life.

8. **Tampa Scale for Kinesiophobia (TSK)**: self-report measure for assessing fear of movement and injury in back pain patients.

9. **Depression (PROMIS)**: 8-item short form PROMIS depression scale. Participants who have severe depression will be referred to the crisis center re:solve Network within Allegheny County, or other crisis centers outside of Allegheny County.

10. **Credibility/Expectancy Questionnaire (CEQ)**: patient's perceived credibility about the treatment and how much expectation there is that the treatment will be helpful.

6. RANDOMIZATION

The randomization will be performed *after* the baseline assessment. The study coordinator will carry out the randomization through web-based computer system used in this study. Randomization scheme is based on a minimization algorithm proposed by Stigsby and Taves[i]. The approach is to balance on multiple baseline prognostic factors using a rank-based method. The variables used will be baseline Swiss Spinal Stenosis Score (SSS), baseline Shuttle Walk Test (SWT), age and Credibility Expectancy Questionnaire (CEQ) score.

7. MASKING

While the treatment assignments obviously cannot be masked to the patient, all clinician-assessed evaluations post-intervention will be carried out by testers blinded to the patient's group assignment. Many of the outcome variables assessed in this study are patient self-report measures. One advantage of self-report measures is the minimization of tester bias. The performance-based variables in this study include the shuttle-walk test and the SPPB. Both self-report and performance-based variables will be assessed by testers who are not involved in the patient's treatment and are blinded to the treatment assignment. Since randomization will be performed after baseline assessment, the variables of physical exam performed at baseline to be explored as potential predictors of outcome will not be influenced by tester bias.

8. STUDY INTERVENTIONS

8.1. Usual Medical Care

Personnel performing the procedures: Dr. Ronald Glick, MD and/or Research Coordinator.
Location of the procedures: UPMC Center for Integrative Medicine, UPMC Shadyside.
Duration of the procedures: Three 30 minute examination visits over a period of approximately 6 weeks.

Procedures:
Subjects will see a board certified physical medicine and rehabilitation physician for a history and examination, after which a determination is made about a course of treatment that is individualized to the needs of each patient. The physician is permitted to prescribe any of the following oral medications, based upon the individual clinical presentation of each subject:

- Non-steroidal anti-inflammatory: ibuprofen, celocoxib, or diclofenac/misoprostol
- Adjunctive analgesics: acetaminophen, tramadol, or pregabalin, gabapentin
- Antidepressant agents: nortriptyline, duloxetine, sertraline, trazodone, or mirtazapine

The physician also has the option to refer subjects for epidural steroid injections, which can be performed at a number of locations in Pittsburgh, including UPMC hospitals or out-patient centers. If recommended, epidurals are standard of care procedures and will be paid for by the study through an Institutional Account. In addition to oral medications and/or epidural injections, all subjects are given advice to stay active and a basic posture advice.

Subjects are asked about treatment compliance, side effects and adverse events at each visit, and complete the Swiss Spinal Stenosis Questionnaire and Oswestry Disability Index at mid-treatment (visit 2).

8.2. Community-based Group Exercise

Personnel performing the procedures: Senior physical fitness instructors at the participating community centers.
Location of the procedures: Jewish Community Center (JCC) in Squirrel Hill or the Vintage Center in East Liberty, both local community senior centers which cater to the needs of older adults.
Duration of the procedures: Approximately 2 exercise classes per week (45 minutes each) for 6 weeks, for a total of 12 classes.

Procedures:
Subjects will attend group exercise classes designed specifically for older adults and taught by senior physical fitness instructors. Participants can make a choice between a class that provides chairs for seated or standing support or another class for people who are comfortable standing without any support. Each class consists of a variety of exercises designed to increase muscular strength, range of movement, and activity for daily living skills. There is a minimal amount of low impact cardiovascular exercise combined with the use of small hand-held weights, elastic tubing and gym balls for gentle resistance exercise.

The participants in these classes are monitored carefully for any signs of physical discomfort and are told to go at their own pace, resting whenever necessary. No specific body region is targeted with these exercise classes. The goal is to provide participants with gentle strength, balance, and generalized fitness training for the entire body. Some of the specific exercises included in these classes are: ankle/wrist rotations, partial squats, leg and knee extension/flexion, elastic tubing for strength training of the upper arm and chest muscles, coordination drills with a gym ball such as bouncing, throwing and catching.

Subjects are asked about treatment compliance, side effects and adverse events, and complete the Swiss Spinal Stenosis Questionnaire and Oswestry Disability Index at mid-treatment (at approximately 3 weeks).

8.3. Clinic-Based Manual Therapy and Rehabilitative Exercise

Personnel performing the procedures: Licensed physical therapists and/or chiropractors who have at least 5 years of clinical experience with manual therapy and rehabilitative exercise procedures.
Location of the procedures: Physical Therapy Clinical and Translational Research Center (PT-CTRC) which is an outpatient facility located on the campus of the University of Pittsburgh.
Duration of the procedures: Approximately 2 treatment sessions per week (45 minutes each) for 6 weeks, for a total of 12 visits.

Procedures:
This group of subjects are treated with a combination of manual therapy and rehabilitative exercise procedures that are well established in the physical therapy and chiropractic literature. A detailed written treatment protocol and clinical decision tree will be reviewed with each clinician, with the chiropractic co-investigator and

consultant providing training sessions before recruitment to ensure treatment consistency. Treatment sessions include a pragmatic use of the individual methods listed below:

- Warm-up on a stationary exercise bicycle; time is individualized to tolerance of each subject
- Distraction-manipulation: this is a form of manually-assisted segmental lumbar traction manipulation using a specialized treatment table
- Neural mobilization: this performed with the patient supine, applying repeated ankle dorsiflexion with the leg extended to generate tension-mobilization of the lumbosacral nerve roots
- Hip, sacroiliac and lumbar facet mobilizations: these are standard manual mobilizations applied to improve segmental mobility in these joints
- Soft tissue mobilizations of lumbopelvic and lower extremity muscles: these include deep pressure over taut bands/myofascial trigger points and post-isometric (contract-relax) stretching techniques
- Self-stretching: patients are taught how to perform neural mobilization and self-stretches for home use
- Spinal stabilization exercise: these include exercises for core stability and spinal range of motion

Subjects are asked about treatment compliance, side effects and adverse events at each visit, and complete the Swiss Spinal Stenosis Questionnaire and Oswestry Disability Index at mid-treatment (approximately 3 weeks).

9. FOLLOW-UP VISITS

All participants will be asked to return to the PT-CTRC for two follow-up evaluations at approximately 2 weeks (Follow-Up 2 months) and 4 months (Follow-Up 6 months) following the 6-week treatment phase. Primary and secondary outcome measures and questionnaire data will be repeated. Each visit will take approximately one hour.

Questionnaires include self report measures of pain and physical function, and other psychosocial measures:

1. Swiss Spinal Stenosis Questionnaire (SSS)
2. Activities Specific Balance Confidence (ABC) Scale
3. Falls History
4. Oswestry Low Back Pain Disability Index
5. The RAND 36-Item Health Survey
6. Global Walking and Mobility Items
7. Patient Global Index of Change (PGIC) and Patient Satisfaction assess patients level of improvement and satisfaction with the treatment.

Physical Exams will be performed by licensed Physical Therapists or Doctors of Chiropractic and will include the following:

1. Shuttle Walk Test (SWT)

2. Short Physical Performance Battery (SPPB)
3. SenseWear Armband

10. ADVERSE EVENTS

10.1. Management

The occurrence of adverse events is monitored for each subject on an ongoing basis throughout the study. Reporting of adverse events in the context of the proposed program of research occurs according to the following definitions:

- Serious. This adverse event is fatal or life-threatening; requires hospitalization, or produces a disability.
- Moderate or greater severity. This adverse event requires medical evaluation and/or medical treatment; or is a serious adverse reaction.
- Unexpected. This event is not identified in nature, severity or frequency in the IRB-approved research protocol or informed consent document.
- Associated with the research intervention. There is a reasonable possibility that this event may have been caused by the research intervention (i.e., a causal relationship between the event and research intervention cannot be ruled out by the investigators).

10.2. Report

All adverse events that are (a) unexpected; (b) of moderate or greater severity; and (c) associated with the research intervention are reported to the IRB. In the case of a serious adverse event, an emergency meeting of the investigative team is called. At the time of this meeting, a determination is made as to whether the trial should be prematurely interrupted. Expected adverse events; unexpected adverse events of minor severity; or adverse events which are determined by the PI to be unrelated to the research intervention are not reported to the IRB. These events are reported to PCORI during the annual report.

All adverse events are reported according to the following timeline: If the event is fatal or life-threatening, the report to the IRB and the PCORI occurs within 24 hours of the event. If the event is unexpected, and of moderate or greater severity (but not fatal or life-threatening), and associated with the research intervention, it is reported to the IRB and the PCORI within 10 calendar days of the reaction. The IRB and the PCORI are also notified as soon as possible of major disputes between the PI and/or project staff and a research subject or between research investigators (including research staff) involved in the proposed program of research if the resolution of the dispute is or will be problematic. If an unexpected adverse event occurs, the PIs re-assess the risk/benefit ratio of the study and submit any modifications deemed necessary to the IRB and the PCORI for approval.

11. STUDY COMPENSATION

Subjects are compensated for their time during treatment (up to $120) and at both follow up visits ($60 each).

12. SAMPLE SIZE JUSTIFICATION

Power analysis: A sample size of 150 subjects (n=50 per group) equates to over 80% power to detect a difference of 3.6 points on the SSS score (with a SD of 6.1 based upon preliminary data). In addition to these tests, the given sample size also yields sufficient power to fit a regression model that detects a statistical difference in the proportion of variability explained. More specifically, a sample size of 150 achieves 81% power to detect an R-square of 5% attributed to two independent variables (representing treatment) and assuming the control variables account for an additional 20% of the variability. We will recruit an additional 30 subjects to account for an anticipated drop-out rate of 15%.

13. DATA ANALYSIS

13.1. Descriptive Statistics

All primary analyses will be preceded by a descriptive analysis of baseline and outcome measures. Summary statistics will be presented both for the overall study cohort and stratified by treatment group. Although we expect that the randomization procedure will balance the treatment groups for all predictors, the significance of any imbalance will be assessed either using analysis of variance (for continuous measures) or chi-square test (for categorical measures). Descriptive statistics and visual summaries will be used to assess assumptions such a normality and linear association between outcomes and predictors of interest. If assumptions are not met, data transformations or more robust statistical procedures will be used.

13.2. Analytic Statistics

We will perform a number of statistical analyses of data obtained from the following 3 comparison groups in this trial:

- Group 1: Usual medical care
- Group 2: Community-based group exercise classes
- Group 3: Clinic-based manual therapy and rehabilitative exercise

Primary Aim: LSS subjects in Groups 2 and 3 will demonstrate greater improvement in self-reported pain/function and walking performance as compared to Group 1.

Statistical analysis 1**:** The primary outcome variables for this hypothesis are Swiss Spinal Stenosis (SSS) score and Shuttle Walk Test (SWT) measured post-intervention at 2 months, and both will be analyzed similarly. We will also assess these outcomes at 6 months. The outcome and all baseline characteristics will be summarized separately by treatment group. Analysis of Variance (ANOVA) will be used to assess the unadjusted association between the outcome variable and treatment groups. A multivariable linear regression model will be used to assess the significance of treatment for each group while adjusting for baseline variables that are at least marginally significant in the unadjusted models ($p<0.10$) and other variables that are selected a-priori for clinical significance (e.g. age). All analysis for treatment group comparisons will use an intention-to-treat approach.

Statistical analysis 2**:** We will also perform a responder analysis using dichotomous outcomes, consistent with the recommendations published by the Initiative on Methods, Measurement, and Pain Assessment in Clinical Trials (IMMPACT). Our responder analysis will compare the percentages of subjects who achieve meaningful outcomes between treatment groups on SSS score and SWT (distance walked). Per IMMPACT recommendations, subjects who achieve at least 30% and 50% decreases in outcome measures are considered to show "moderate improvement" and "substantial improvement", respectively. Differences in the proportion of responders versus non-responders between the groups will be assessed using Chi-square tests. Multiple logistic regression models will be used to assess the association of each of these dichotomized outcomes (>30% followed by >50%) by group, adjusted for covariates. The covariates will be chosen based upon univariate logistic regression ($p<0.10$) and clinical relevance.

Secondary Aim: Subjects in Groups 2 and 3 will demonstrate a greater change in physical activity compared to Group 1.

Statistical analysis 1**:** The primary outcome variable for this hypothesis is physical activity data captured by the SenseWear Armband measured post-intervention at 2 months. To compare changes in physical activity levels between the three treatment groups linear regression analysis will be used. Univariate regression models will be used to assess the unadjusted association between the outcome variable and treatment groups. A multi-variable linear regression model will be used to assess the significance of treatment for each group while adjusting for baseline variables that are at least marginally significant ($p<0.10$) and other variables that are selected a-priori for clinical significance. All analysis for treatment group comparisons will use an intention-to-treat approach.

Statistical analysis 2**: Responder analysis**

Exploratory Aim 1: For all three treatment groups there will be a low incidence of adverse events and similar treatment adherence rates. The 6-month attrition rate, number of falls, will be lower in Groups 2 and 3 compared to Group 1.

Statistical analysis**:** Descriptive analysis of 6-month attrition rates, number of adverse events, treatment adherence rates, number of falls, will be conducted using simple frequencies and contingency tables for associations with treatment group. Although these analyses will focus on descriptive summaries and judgments of clinical important differences, chi-squared or Fisher's exact tests will also be conducted to quantify the corresponding significance levels.

Exploratory Aim 2: Heterogeneity of treatment effect. A group of baseline physical, psychosocial, and demographic measures will be associated with treatment response and non-response in each group. We expect baseline predictors of treatment response to be different in Group 1 as compared to Groups 2 and 3.

Statistical analysis: The responder analysis will compare the percentages of subjects who achieve meaningful outcomes between treatment groups on SSS score and SWT (distance walked). Each subject will be classified as a responder or non-responder based on a minimum change score of 30%, thus yielding a binary outcome. Baseline variables will be summarized separately for responders and non-responders. Unadjusted odds ratios will be estimated using univariate logistic regression, and (in the same manner as previously described for linear regression) multivariable logistic regression models will be utilized to adjust for multiple baseline factors, including treatment group, and psychosocial and demographic measures. We will also test two-way interactions between

treatment group and baseline predictors versus the final main effects model. The multiple regression models will be limited to no more than one predictor per event in the logistic model; if more variables are significant, the model will be limited to the most significant variables, after adjusting for those deemed a-priori to be clinically significant.

14. DROPOUT AND MISSING DATA

If a substantial number of subjects is found to have missing data, we will first determine the causes of why data are missing. These possibilities could be missing completely at random, missing at random, or not missing at random. We will compare characteristics between subjects that have observed data at follow up with those that have missing values. In the case where data is missing at random, multiple imputation will be used to substitute for the missing values using a pre-specified model. Once the imputed datasets have been created (we will use M = 5 imputations) the proposed analysis will be performed on each of the new datasets and the estimates of interest will be combined.

In the case of not missing at random, the missing data mechanism must be modeled in order to obtain unbiased parameter estimates. The missing not at random assumes the missingness is directly related to the value of the missing data. In that case, methods specifically designed to handle the missing not at random mechanism will be used such as Pattern Mixture Models (PMM). With the PMM, we can simultaneously model whether a subject is a completer (has the data at both time points) versus a non-completer (does not have the data at both time points). We would include an indicator variable for the non-completers as a predictor in the regression model of our outcome measure and examine its interaction with the study covariates.

15. RECORD KEEPING

The majority of the forms and questionnaires used in this study are entered directly into the database. The hard copy of the forms completed on paper are being stored in subject's file and kept in a locked file cabinet in the trial coordinator's office. Only study personnel has access to these files.

A copy of the Informed Consent signed by the subject and by the trial coordinator is kept in a separate locked file cabinet in the trial coordinator's office, as well as the Contact Information form, Telephone screening form, and any medical record data provided by the subject containing identifiable information.

The database is web-based for direct data entry, where subjects are identified by a study ID, and no personally identifiable information is stored in it or used in any of the analyses.

16. DATA MANAGEMENT PLAN

Data management is overseen by the PI, Research Coordinator, and is coordinated by the Office of Academic Computing (OAC), University of Pittsburgh. The OAC created an electronic System for Data Management [WebDataXpress (WDX)] for data collection, tracking, follow-up, reporting, and analysis. The WDX system includes verifying the data, out of range data checks, and repeated evaluation of data process, eliminating the possibility of most incorrect entries and preventing extensive recoding and cleaning by the statistician.

The primary method of data collection is through the database. However, if access to the internet is disrupted, paper forms are available to ensure data collection. All data collected in paper forms are stored in the subject's research chart identified by their ID. In this case, coordinator contacts the database programmers via telephone to obtain the subject's group assignment.

17. QUALITY ASSURANCE

To ensure data quality and integrity we are using standard methods of data collection and recording, have formal staff workshops on research integrity, document computer operations and data editing procedures, and have regular meetings with project staff to review any changes in procedure. The electronic forms are maintained by OAC on a local network in a relational database. All files are backed-up daily and archived weekly.

18. HUMAN SUBJECTS

18.1. Institutional Review Board (IRB) Review and Inform Consent

This protocol and the informed consent document and any subsequent modifications will be reviewed and approved by the University of Pittsburgh IRB (PRO12120422). A signed informed consent form is obtained from all subjects. The consent form describes the purpose of the study, the procedures to be followed, and the risks and benefits of participation. Subjects will be informed that they are free to withdraw from the study at any time. A copy of the signed informed consent form is given to the subject.

18.2. Potential Risks/Risk Management

<u>Risks associated with Interviews and Questionnaires:</u> Completing the questionnaires may cause subjects to feel uncomfortable when answering questions of a personal nature. To minimize this risk, subjects may choose not to answer any of the questions regardless of reason.

<u>Risks associated with baseline physical examination</u>: The risks associated with the physical examination procedures may include temporary muscle soreness or tripping and falling during testing and/or exercises. During testing and training, risks of tripping and falling will be minimized by providing direct stand-by supervision by the research staff. In addition, the exclusion criteria used in this study provides that individuals who are prone to falling, or have a neurodegenerative disorder will not be participating in the study. When engaging in exercise, there is a rare risk that subjects may experience chest pain, dizziness, shortness of breath, heart attack, or stroke.

<u>Risks associated with manual therapy and exercise:</u> The most common side effects of manual therapy and exercise include mild discomfort and soreness in the lower back, hip, or buttock regions that usually goes away within a day or two. More serious complications are extremely rare and their association with spinal manipulation is debated. These complications include nerve damage, injuries to spinal discs, and spinal fractures. The chance of serious complications is estimated to be 1 per million manipulations of the lower back. When engaging in exercise, there is a rare risk that subjects may experience chest pain, dizziness, shortness of

breath, heart attack, or stroke. To minimize this risk, participants are instructed not to exert themselves beyond their level of physical comfort and that they should inform the exercise instructor or clinician about any of these symptoms.

Risks associated with usual medical care: The most common risks are potential side effects to various medications. All subjects in this treatment arm will be examined and monitored by a physician who is board certified in physical medicine and rehabilitation, and psychiatry. He will conduct a thorough case history and carefully ask patients about their current medications, as well as any side effects or allergies they've had with previous medications. He will also monitor all patients for any medication side effects and discontinue or change any medications that are not well tolerated.

Pain medications may include Tylenol, tramadol, pregabalin, or gabapentin. There are relatively little side effects associated with the normal use of Tylenol; the most serious and rare side effect of Tylenol use is liver damage due to large doses, chronic use, or taking Tylenol along with large amounts of alcohol or other drugs that may damage the liver. Common side effects of tramadol include: Constipation, dizziness, headache, nausea, vomiting, decreased lung function. Common side effects of pregabalin include: dizziness, drowsiness, loss of balance, dry mouth, constipation, edema, tremors, blurred vision, weight gain. Common side effects of gabapentin include: Drowsiness, dizziness, low energy, muscle pain, fluid retention, blurred vision.

Anti-inflammatory medications may include any of the following: ibuprofen, naproxen, or celecoxib. Common side effects of these medications include: Tinnitus, rash, hives, water retention, drowsiness, dizziness, headache, nausea, heart burn, stomach cramps, swelling.

Mood/sleep medications may include any of the following: Nortriptylin, Trazodone, Duloxetine or Venlafaxine. Common side effects of these medications are: dry mouth, low blood pressure, constipation, drowsiness, dizziness, low energy, nausea, vomiting, headache, anxiety, sexual problems, loss of appetite, insomnia, diarrhea.

If the doctor recommends an epidural injection, there are other potential side effects and risks, most of which are considered rare. Post-injection infections from the needle puncture can occur, but are very rare. Post-injection bleeding is another rare risk, which is more common in patients taking blood thinner medications. Direct damage to a spinal nerve from the needle is another rare risk, and associated only with one type of nerve block procedure. Temporary reduction of bladder tone from the anesthetic effect on the nerves is also possible. In rare cases there may be a leakage of spinal fluid from the needle puncture associated with post-injection headache, requiring a blood patch to stop the leakage.

18.3. Privacy

All baseline and follow-up research activities will take place in a private room at the Physical Therapy Clinical and Translational Research Center. The data collected will be limited to information necessary to achieve the aims of this research. PT treatment sessions take place in an open treatment room with the necessary equipment due to the nature of the treatment. Participants are not specifically identified as part of this research study; but note that these treatment sessions take place in the PT-CTRC and all individuals receiving therapy in this location are participating in some type of research protocol.

Participants in the Usual Medical Care group will be seen at the UPMC Center for Integrative Medicine in a private room. They will not be identified as research participants in the reception room or waiting areas.

Participants in the group exercise intervention will be provided with a membership to the JCC or Vintage Center and will be treated in the same manner as other regular members of the facility. They will not be identified in their exercise classes in any way as research participants. The administrative teams at the JCC and Vintage Center have completed the approved CTSI Community Partner Research Ethics training program with the PI.

18.4. Confidentiality

Patient confidentiality is maintained throughout the study. The risk of breaching subject confidentiality is minimized by using a web-based system of data entry. The data is directly entered into a computer at the time of the interviews. A relational database is stored on a local network where only select research team members have access. All study subjects are assigned unique study identifiers that appear on all data collection instruments, documents, and files used in the statistical analysis and manuscript preparation. Any items containing identifiable information, such as patient names on consent forms, will be stored in a locked file cabinet in a separate location from data forms. Only limited team members have access to personal information needed for tracking and informed consent. No personal information concerning study participants will be released without their written consent.

18.5. Potential Benefits of the Proposed Research to Human Subjects and Others

All of the treatments provided in the three arms of this study have the potential to directly benefit the research participants. The oral medications and spinal injections are commonly used by physical medicine and rehabilitation physicians to treat patients with lumbar spinal stenosis, and have the potential of relieving pain and improving function. Community based group exercise classes - such as the popular Silver Sneakers program - have been taught to older adults around the country for many years with good success. Although not specifically designed for patients with lumbar spinal stenosis, these group exercise classes have the potential of improving physical fitness, balance and walking performance in those who participate. Clinic based physical therapy and chiropractic treatment have been shown in several observational studies to benefit some patients with lumbar spinal stenosis.

18.6. Importance of the Knowledge to be Gained

Many patients with LSS are poor candidates for spine surgery due to high risk from co-morbidities such as diabetes, obesity and cardiovascular disease. In addition, a large number of LSS patients with mild or moderate symptoms and functional limitation simply do not require surgical intervention. At this point, little is known about their chances of improving with non-surgical treatment. Patients who cannot - or choose not - to have surgery need more information to help make informed decisions about the effectiveness of the various non-surgical treatment options available to them for managing their LSS symptoms.

This is the basis for the Primary Aim of our study: to compare the clinical effectiveness of three pragmatic non-surgical treatment approaches.

APPENDIX *(documents from appendix are available upon request)*

A. **Study Forms**
B. **Informed Consent**
C. **Study Advertisement**

Clinical Intervention Study Protocol

A Comparison of Non-Surgical Treatment Methods for Patients with Lumbar Spinal Stenosis

Principal Investigator:

Michael J. Schneider, DC, PhD

Supported by:

Patient Centered Outcomes Research Institute (PCORI)

Version 1.3

November 1, 2017

CONTENTS

AMENDMENTS 4
1. STUDY OBJECTIVES 4
1.1. Primary Aim 7
1.2. Secondary Aim 7
1.3. Exploratory Aim 1 7
1.4. Exploratory Aim 2 7
2. BACKGROUND 8
3. STUDY DESIGN 9
3.1. Overview of Study Design 9
3.2. Summary of Study Recruitment and Screening Procedures 12
4. SELECTION AND ENROLLMENT OF SUBJECTS 12
4.1. Inclusion/Exclusion Criteria 12
4.2. Telephone Screening Process and In-person Eligibility Screening Visit Scheduling 13
4.3. Baseline Screening and Exam 13
5. BASELINE TESTING 14
5.1. Clinical Examination 14
6. RANDOMIZATION 19
7. MASKING 19
8. STUDY INTERVENTIONS 19
8.1. Usual Medical Care 19
8.2. Community-based Group Exercise 20
8.3. Clinic-Based Manual Therapy and Rehabilitative Exercise 20
9. FOLLOW-UP VISITS 21
10. ADVERSE EVENTS 22
10.1. Management 22
10.2. Report 22
11. STUDY COMPENSATION 23
13. DATA ANALYSIS 23
13.1. Descriptive Statistics 23
13.1. Analytic Statistics 23

14. DROPOUT AND MISSING DATA 25
15. RECORD KEEPING 25
16. DATA MANAGEMENT PLAN 26
17. QUALITY ASSURANCE 26
18. HUMAN SUBJECTS 26
18.1. Institutional Review Board (IRB) Review and Inform Consent 26
18.2. Potential Risks/Risk Management 26
18.3. Privacy 28
18.4. Confidentiality 28
18.5. Potential Benefits of the Proposed Research to Human Subjects and Others 28
18.6. Importance of the Knowledge to be Gained 28
APPENDIX *(documents from appendix are available upon request)* 29
A. Study Forms 29
B. Informed Consent 29
C. Study Advertisement 29

Table of Figures

Figure 1- Study Design 8

Table of Tables

Table 1. Measures completed at each time point 9

AMENDMENTS

Protocol Version	Approved Date
Original Protocol 1.0	April 1, 2013
Version 1.1	May 1, 2013
Version 1.2	September 1, 2015
Version 1.3	November 1, 2017

Amendment 1 v 1.1: April 2013

The overall reason for amendment: Change in performance based outcome measure prior to subject enrollment.

Protocol Changes: Changed the Shuttle Walk Test (SWT) to the Self-Paced Walk Test (SPWT).

Reason for Change: We substituted the Self-Paced Walking Test (SPWT) for the Shuttle Walk Test (SWT) and notified PCORI of this change prior to subject enrollment. This change in our choice of walking performance measure occurred for 2 reasons. First, the developer of the SPWT consulted with us about our research design and presented evidence about the reliability and accuracy of the SPWT. Secondly, feedback from focus groups with stenosis patients indicated that the SPWT was a more patient-centered outcome that replicated real-life walking performance.

Amendment 2 v 1.1: April 2013

The overall reason for amendment: Change in Usual Medical Care protocol prior to subject enrollment.

Protocol Changes: Eliminated from permitted prescribed medications: tramadol and pregabalin

Reason for Change: Prior to initiation of the study, Tramadol was reclassified to Schedule IV controlled substance and we wanted to avoid prescribing any type of opioid medication. We eliminated pregabalin from our list of permitted medications because we thought that it would be cost prohibitive considering that once subjects completed the 6-week treatment period, the study would no longer cover the cost of the prescription.

Amendment 3 v.1.1: April 2013

The overall reason for amendment: Change in inclusion criteria prior to subject enrollment.

Protocol Changes: Corrected age for inclusion to be greater than or equal to 60 years.

Reason for Change: Correction.

Amendment 4 v 1.1: April 2013

The overall reason for amendment: Change to inclusion criteria prior to subject enrollment.

Protocol Changes: Changed walking ability inclusion from 100 feet to 50 feet without an assistive device.

Reason for Change: After consultation with our physical medicine and rehabilitation consultant, he suggested that walking 50 feet without a cane, walker or other assistive device would be a more reasonable inclusion criterion. He was concerned that an inclusion criterion of 100 feet was overly restrictive.

Amendment 5 v 1.1: April 2013

The overall reason for amendment: Change to exclusion criteria prior to subject enrollment.

Protocol Changes: Added exclusion criteria severe hypertension; resting blood pressure of greater than 200 mm Hg systolic or 110 mm Hg diastolic.

Reason for Change: We decided to adhere to the American College of Sports Medicine guidelines for participation in exercise, which state that a resting blood pressure of greater than 200 mm Hg systolic or 110 mm Hg diastolic is a contraindication to exercise.

Amendment 6 v 1.1: April 2013

The overall reason for amendment: Change to randomization schema.

Protocol Changes: Removed Credibility Expectancy Questionnaire (CEQ) score as a randomization variable.

Reason for Change: Our biostatistician reviewed baseline data from other clinical trials on low back pain and noticed a curious pattern; most of the CEQ scores were relatively high and there was a lack of variation in CEQ scores by group assignment. We hypothesized that by virtue of agreeing to be randomized, that research participants have relatively high expectations that any of the treatments used in the study may be helpful. We decided to continue to collect CEQ scores at baseline for an analysis of baseline characteristics by group, but to remove this as a randomization variable. We decided to only use 3 key baseline variables for our adaptive allocation randomization algorithm: age, baseline Swiss Spinal Stenosis questionnaire score, baseline Oswestry Disability Index score.

Amendment 7 v 1.2: September 1, 2015

The overall reason for amendment: Increase in sample size at the request of PCORI.

Protocol Changes: Increased total sample size from 180 to 260.

Reason for Change: Due to an early unanticipated success in recruitment and enrollment, the PCORI program officer asked us to continue recruitment for an additional 6 months. We subsequently received supplemental funding from PCORI, and continued recruitment efforts which led to a final sample size (N=259). No interim data

analysis was performed after reaching our original sample size of N=180; only a single data analysis was conducted using data from the final sample size of N=259.

Amendment 8 v 1.3: November 1, 2017

The overall reason for amendment: Modification in statistical plan.

Protocol Changes: We had originally planned to handle missing data by using multiple imputations and running sensitivity analyses. This was changed to the use of linear mixed models to follow an intention-to-treat principle.

Reason for Change: after reviewing the data our biostatistician wanted to be able to perform an intention-to-treat analysis and include all subjects who dropped out after randomization, but never had any treatment. Therefore she suggested using linear mixed models, which use all available data; if a subject has a measurement at one time-point and the rest of the data is missing, that subject is still used in the analysis. Therefore, these mixed models included data from all participants who had a baseline examination and were randomized (N=259).

Amendment 9 v 1.3: November 1, 2017

The overall reason for amendment: Modification in statistical plan.

Protocol Changes: Our original primary analysis was a multivariable linear regression model to assess the significance of treatment for each group while adjusting for baseline variables. We originally proposed to use step-wise elimination models using the variables that were least marginally significant in the unadjusted models ($p<0.10$) and other variables that are selected a-priori for clinical significance (e.g. age). This was modified to force enter only the 3 baseline variables used in the randomization algorithm.

Reason for Change: Our biostatistician thought it would be a more accurate analysis to use linear mixed models and only include in the models those 3 baseline variables used in our randomization algorithm (age, baseline Swiss Spinal Stenosis Questionnaire score, baseline Oswestry score).

1. STUDY OBJECTIVES

This study is a pragmatic comparative effectiveness study of three types of non-surgical treatment options for patients with Lumbar Spinal Stenosis (LSS). The main goal is to provide evidence to inform the choices between three types of non-surgical treatment options for patients with LSS. The three comparison groups in this trial are:

- Group 1: Usual medical care
- Group 2: Community-based group exercise classes
- Group 3: Clinic-based manual therapy and rehabilitative exercise

1.1. Primary Aim

To compare the clinical outcomes between these 3 treatment groups using two validated primary outcome measures of pain and physical function; Swiss Spinal Stenosis questionnaire (self-report measure of pain/function) and Shuttle Walk Test (performance-based measure of function).

- Hypothesis: LSS subjects in Groups 2 and 3 will demonstrate greater improvement in self-reported pain/function and walking performance as compared to Group 1.

1.2. Secondary Aim

To compare changes in physical activity between these 3 treatment groups using the SenseWear Armband (real-time measure).

- Hypothesis: Subjects in Groups 2 and 3 will demonstrate a greater change in physical activity compared to Group 1.

1.3. Exploratory Aim 1

To explore relationships between 6-month attrition rates, number of adverse events, adherence rates, number of falls, and co-interventions across treatment groups.

- Hypothesis: For all 3 treatment groups there will be a low incidence of adverse events and similar treatment adherence rates. The 6-month attrition rate, number of falls, and co-interventions will be lower in Groups 2 and 3 compared to Group 1.

1.4. Exploratory Aim 2

To explore potential baseline predictors of treatment response in each of the 3 treatment groups.

- Hypothesis: A group of baseline physical, psychosocial, and demographic measures will be associated with treatment response and non-response in each group. We expect baseline predictors of treatment response to be different in Group 1 as compared to Groups 2 and 3.

2. BACKGROUND

Lumbar spinal stenosis (LSS) is the most common reason for spinal surgery in older adults. It has been shown that surgery is effective for severe cases of LSS, but many patients with mild to moderate symptoms are not surgical candidates. These patients and their providers are seeking effective non-surgical treatment methods to manage their symptoms; yet there is a serious lack of comparative effectiveness research in this area.

LSS is a degenerative arthritic disease of the spine which is often associated with significant functional limitations of walking and disability. Radiographic and clinical data from the Framingham cross-sectional study reports the prevalence of degenerative lumbar stenosis at 30%. The hallmark symptom of the clinical syndrome of LSS is significant leg pain. The leg pain is typically worse with walking, which often leads to a dramatic impairment in ambulation and increased risk of falling; comparable to patients with severe knee osteoarthritis. Decreased ambulation leads to decreased physical activity, which can negatively impact overall health. Limitation of physical activity is associated with obesity, heart disease, peripheral artery disease, diabetes, and cognitive decline. Impairment of walking ability can also have a negative impact on the ability of older adults to stay in the workforce and live independently.

The findings of our research may have a strong impact on the efficiency of patient care in several ways. As noted previously, LSS is the most common reason for spinal surgery in older adults, with a lack of clear guidelines about the best choices for non-surgical care options. Patients, providers, payers and policy makers are all left in a quandary about how to make informed decisions about their respective management of LSS. Research evidence is urgently needed to inform all of these stakeholders about the clinical benefits and risks of the common non-surgical approaches being compared in this proposed study.

Patients with LSS need more information about the benefits and risks of the non-surgical treatment options being offered to them. Primary care physicians and surgeons who are caring for LSS patients also report that they simply don’t have enough good evidence to inform their choices about which non-surgical treatments to recommend for their patients. This includes the spine surgeons and neurosurgeon who have provided the PI with letters of support for this study. They have indicated being unsure of the best clinical approach to take with LSS patients for whom they have determined surgery is not warranted or contraindicated. This proposed study should help to provide both patients and providers with meaningful information to better guide their treatment choices.

This research will also provide evidence that may influence changes at the level of payers and policy makers. One of the key stakeholders cooperating with this proposed study is the University of Pittsburgh Medical Center (UPMC) Health Plan. The UPMC Health Plan recently instituted a policy mandating a three-month course of chiropractic, physical therapy, and oral medication before authorization of any spine surgery for chronic back pain, including spinal stenosis. The senior medical director indicated that internal research at the UPMC Health Plan uncovered the fact that less than 50% of patients undergoing spine surgery had been treated by a physical therapist or chiropractor prior to their surgery. The percentage of lumbar spinal stenosis patients receiving non-surgical treatment prior to surgical intervention was even lower. These low utilization rates are of particular

concern to the Health Plan, because it implies that LSS patients are not accessing many potentially beneficial non-surgical options.

It is also possible that the results of our study will provide enough evidence to stimulate other larger payers and policy makers to follow the lead of the UPMC Health Plan, and provide incentives to patients and providers to explore a trial of non-surgical treatment before considering spine surgery. In the absence of good evidence, patients with LSS and the providers who care for them are left feeling uncertain about the benefits and risks of group exercise classes, manual therapy and rehabilitative exercise for LSS. We believe the results of our study will remove some of this uncertainty and thereby have a strong impact on health care performance at the level of patients, providers, payers and policy makers.

3. STUDY DESIGN

3.1. Overview of Study Design

This study is a prospective randomized controlled clinical trial of 259 older adults who have both an anatomic diagnosis of LSS and signs and symptoms consistent with a clinical diagnosis of LSS confirmed by clinical examination. Eligible subjects will be randomized into one of three pragmatic treatment groups: 1) usual medical care; 2) community based group exercise; or 3) individualized manual therapy and rehabilitative exercise. All subjects will be treated for a 6-week course of care. Follow-up evaluations will occur at 2 and 6 months after randomization. Figure 1 gives an overview of study design.

Figure 1: Diagram of Study Flow

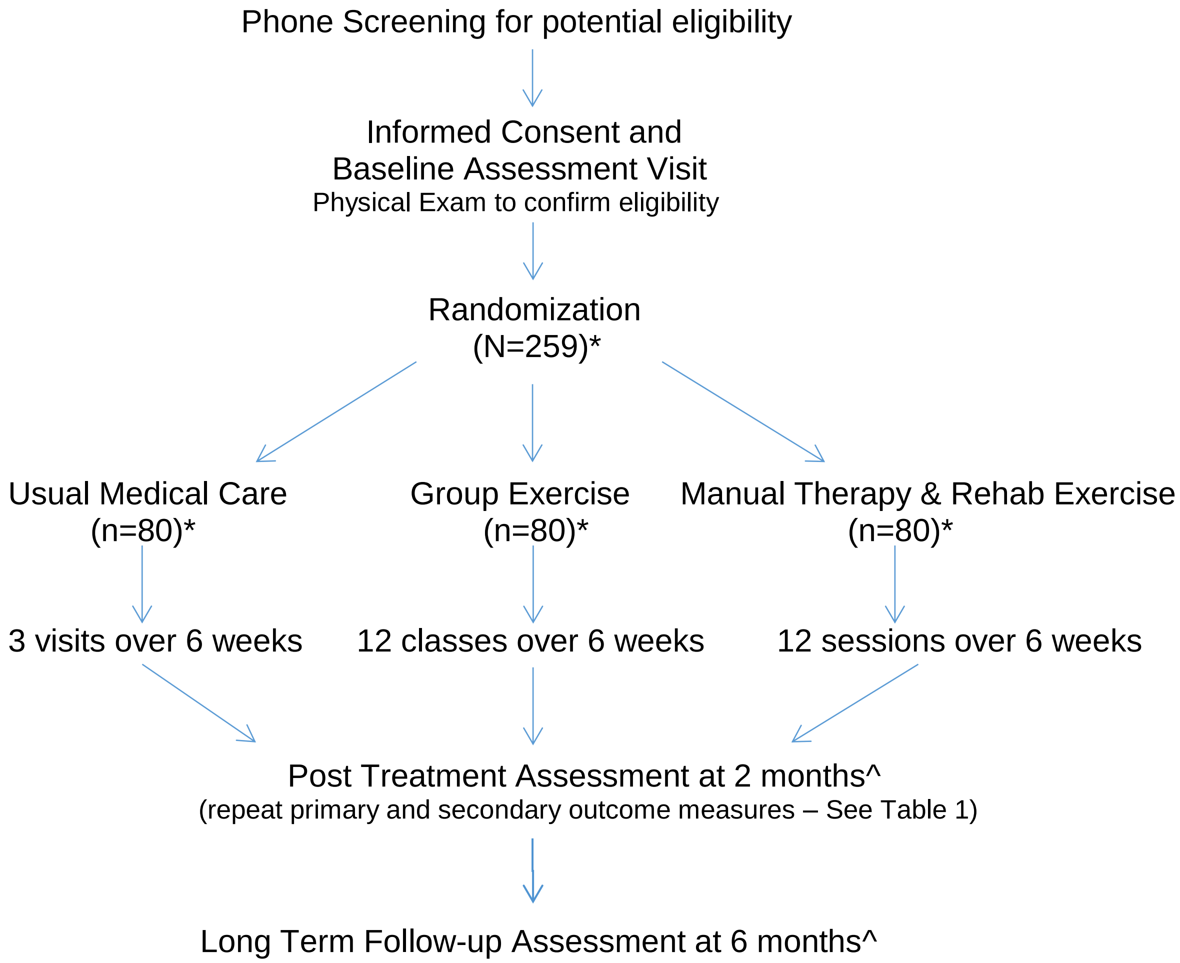

*Sample size of N=259 allows for a less than 10% attrition rate.

^Post treatment and long term follow-up assessments will repeat the primary (SSS, SPWT) and secondary (SWA) outcome measures

Abbreviation Key
SSS = Swiss Spinal Stenosis questionnaire
SPWT = Self-Paced Walk Test
SWA = SenseWear Armband

Table 1: Timeline of Outcome Measures				
Measure	Baseline	Mid-Treatment	Follow Up 2 months	Follow Up 6 months
Primary Outcomes: Physical Function				
Self-report=SSS	x	x	x	x
Performance-based= SPWT	x		x	x
Secondary Outcomes				
Physical Activity Real-time = SWA*	x		x	x
Falls History	x		x	x
Balance Confidence Scale (ABC)	x		x	x
Oswestry	x	x	x	x
Side Effects, Adverse Events		x	x	x
RAND-36	x		x	x
SPPB	x		x	x
PGIC/Satisfaction			x	x
Other Variables				
Demographics	x			
Co-morbidities (MCDI)	x			
Baseline History/Physical Exam	x			
Psychosocial				
Fear (TSK)	x			
Depression (PROMIS)	x			
Expectancy (CEQ)	x			

Abbreviation Key:
SSS=Swiss Spinal Stenosis questionnaire
SPWT=Self-Paced Walk Test
SWA=SenseWear Armband
MCDI=Modified Co-morbidity Disease Index
SPPB= Short Physical Performance Battery
ABI=Ankle Brachial Index
TSK=Tampa Scale for Kinesiophobia
PROMIS= Patient Reported Outcomes Measurement Information System
CEQ=Credibility/Expectancy Questionnaire
PGIC= Patient Global Index of Change

3.2. Summary of Study Recruitment and Screening Procedures

The study uses several recruitment strategies with the intent of enrolling 8 subjects each month. The primary strategy comprises of direct mailings of postcards to adults age 60 and above residing in local neighborhoods.

Additional recruitment strategies include physician referrals, study letters sent to participants of research registries (Clinical and Translational Science Institute -CTSI - Registry and the Pittsburgh Claude D. Pepper Older Americans Independence Center Registry), advertisements on local radio stations, newspapers, and other publications. The study also recruits subjects directly from the Vintage Community Senior Center and the Squirrel Hill Jewish Community Center. Both centers are designated community senior citizen centers by the Allegheny County Area Agency on Aging. The directors of these centers have agreed to assist with the study's recruitment efforts by allowing posters and informational brochures to be placed in their facilities, by sending e-blasts to their respective members, and by posting announcements about the research study in their newsletters.

For all recruitment efforts, we use services from the University of Pittsburgh - University Marketing Communications (UMC). The UMC is a resource available to researchers at the University of Pittsburgh, which has a full-time staff that provides advertising, planning, copywriting, design, and production services and handles reservation of newspaper space.

All recruitment materials have the research coordinator's telephone number so that subjects may contact the research team, if interested in the study. Thus, regardless of the recruitment method, the potential subjects initiate contact with study personnel. All individuals who call to inquire about the study give their verbal consent to undergo telephone screening. Those deemed potentially eligible over the phone will be scheduled for an in-person assessment to confirm eligibility.

4. SELECTION AND ENROLLMENT OF SUBJECTS

4.1. Inclusion/Exclusion Criteria

Inclusion Criteria: Subjects will be **included** in the study if they:

- Are 60 years of age or older

- Have a clinical history of lumbar spinal stenosis (LSS)
- Have imaging (CT or MRI) evidence of LSS
- Have the ability to read/write English and understand instructions
- Have limitation of standing and/or walking tolerance (neurogenic claudication)
- Agree to attend 2 treatments per week for 6 weeks
- Have the ability to engage in mild exercise
- Can walk 50 feet without need for an assistive device
- Are willing to be randomized to one of the 3 treatment groups

Exclusion Criteria: To ensure safety, participants will be **excluded** from the study if they:

- Have a history of metastatic cancer
- Had previous lumbar surgery for LSS; i.e. laminectomy and/or spinal fusion
- Currently have cauda equina symptoms including saddle paresthesia
- Have a history of severe Peripheral Artery Disease and/or Ankle Brachial Index < 0.8 (vascular claudication)
- Have been told by a physician that they should not engage in aerobic exercise
- History of neurologic (e.g., cervical myelopathy, stroke) or neuro-degenerative (e.g., Parkinson's) disease other than LSS that affects the subject's ability to walk
- Cannot complete a self-paced walking test for any reason other than symptoms related to LSS (e.g. chest pain, severe hip arthritis, etc.)
- Systolic blood pressure > 200 mm Hg or Diastolic blood pressure > 110 mm Hg

4.2. Telephone Screening Process and In-person Eligibility Screening Visit Scheduling

All potential research participants recruited to this study will first be screened by telephone for eligibility criteria by research assistants who will be trained to follow a screening script and checklist. The checklist will include the key inclusion/exclusion criteria that can be evaluated by phone. Potential participants who pass the telephone screen will be scheduled for a screening/baseline appointment at the PT-CTRC for further evaluation.

The research coordinator mails or emails a copy of the informed consent document, driving directions to get to the research facility and a letter with general guidelines for the entire visit.

The appointment is confirmed with the subject one day prior to the scheduled visit. The research coordinator must contact the subject to verify if the subject has any questions about the study (after reading the informed consent), remind subject to bring an updated list of medications, discuss transportation and directions, and re-confirm or reschedule the appointment.

4.3. Baseline Screening and Exam

The purpose of the baseline evaluation is to confirm eligibility and collect baseline data. Personnel performing the procedures: Licensed Physical Therapists or Doctors of Chiropractic, and/or Research Coordinator. Location of the procedures: Physical Therapy Clinical & Translational Research Center (PT-CTRC): a component of the University of Pittsburgh Clinical Translational Science Institute (CTSI) and is a state-of-the-art outpatient facility located in Forbes Tower at the University of Pittsburgh main campus.

Duration of the procedures: The Baseline Screening and Exam will take approximately 2 hours.

At the in-person baseline screening visit, the research coordinator explains the study to the potential subject and clarifies any questions the subject might have. The consent form is reviewed using a PowerPoint presentation that explains the key elements. If the subject has no questions or after addressing the subject's concerns, both the coordinator and the subject sign the informed consent (a signed copy is given to the subject and the original is kept in a locked research file).

All subjects are asked to complete a Contact Information Form that is used for compensation purposes (i.e., to assign a pre-paid card to the subject).

The following questionnaires are completed: Demographics, Current Medications

The research coordinator confirms the pre-screening self-reported eligibility/ineligibility criteria.

At any point during the screening process if a subject is deemed ineligible the evaluation is terminated and the subject is compensated for his/her time.

If the subject is deemed eligible for the study, the research coordinator introduces the subject to the tester who continues onto baseline eligibility exam component of this visit.

5. BASELINE TESTING

5.1. Clinical Examination

Baseline symptoms and clinical history These questions are asked verbally while subject is lying supine in preparation for the ABI test:

1. Duration of leg symptoms
2. Duration of back symptoms
3. Predominant pain: legs, low back, equal
4. VAS intensity of leg pain (0-10)
5. VAS intensity of back pain (0-10)
6. Do symptoms improve when bending forward?
7. Do symptoms improve when bending backward?
8. Do symptoms occur when standing up?
9. Is your ability to walk impaired by leg pain?
10. Does your leg pain improve once you sit down and rest?
11. Does leaning forward on an assistive device allow you to walk farther? (shopping cart, cane, walker)
12. Pain extends to: buttock, thigh, calf/foot
13. Do you have urinary incontinence? If yes, does it happen only when walking?
14. Qualitative description of leg symptoms: a) Neurologic: numbness, tingling, pins & needles, burning or b) Pain
15. Have you had hip/groin pain for most days in the past month?

16. Have you had knee pain for most days in the past month?>
17. Do you have morning hip/groin stiffness? < 60 minutes duration?
18. Do you have morning knee stiffness? < 30 minutes duration?
19. Pain present on internal hip rotation? (demonstrate)

2. **Ankle Brachial Index (ABI)**: We will use a standard hand-held Doppler ultrasonic device and sphygmomanometer to record the systolic and diastolic pressures over the dorsal pedis, posterior tibial, and brachial arteries. The ABI is the ratio of the lower to upper extremity systolic pressures, with the normal range being >0.9. We will exclude patients with an ABI <0.8 at the baseline from participation in our study, and will offer to send a report of our findings to their primary care physician for further evaluation. Individuals with a Systolic blood pressure > 200 mm Hg or Diastolic blood pressure > 110 mm Hg will not be eligible to participate and will be referred to their primary care physician for further evaluation.

The subject must lie supine with the socks/shoes off for about 10 minutes below performing the ABI. Make sure the feet are not cold. It is okay to cover the feet with light blanket or sheet while they are acclimating to the room temperature. Use the Doppler unit to find the pulse over the medial malleolus (posterior tibial artery) and then pump up the blood pressure cuff until the sound disappears. Slowly release the pressure and record the systolic pressure as the moment the sound re-appears. The diastolic pressure is recorded when the sound disappears again. Repeat on opposite ankle. Then perform brachial blood pressure readings at both elbows. Record all results directly into the iPad. For small framed patients, use the child-sized cuff if necessary over the ankles. If the cuff is too large, it could lead to an inaccurate reading.

Make sure to confirm that the patient is eligible for the study after completing the ABI procedure. If the iPad tells you the patient is ineligible at this point in the exam, please inform the patient that the results of this test indicate that they need to contact their primary care physician about the possibility of PAD.

2. **Height and weight:** record height in centimeters and weight in kilograms.

3. **Neurological Exam**: consists of 5 tests performed bilaterally. The purpose of the neuro exam is to give the patient a complete physical examination that would typically be performed by a physical medicine and rehabilitation physician or spine surgeon. We will not be excluding patients from the study based upon their neuro exam findings. However, we would alert the patient to any of these findings that we feel should be shared with their primary care physician: positive Babinski, severe loss of muscle function, pinprick or vibration sense.

1) Reflexes: knee and ankle - recorded as Absent, Normal or Hyper. Sluggish or slightly exaggerated reflexes will be collapsed into the category “Normal”. Only clearly hyper-reflexic responses will be recorded as “Hyper”.

2) Vibration sensation: We will use the bio-thesiometer to quantify vibratory sensation at the great toe and medial malleolus. Patients will be familiarized with the sensation by turning the amplitude to maximum and then tested by gradually increasing the amplitude from zero and asking when they first feel the sensation. The lowest of two readings will be recorded.

3) Pinprick sensation exams will be performed over 3 anatomical regions: medial ankle, dorsal foot, and lateral ankle. These areas correspond respectively to the L4, L5, and S1 dermatomal distributions. Record three findings: Absent, Diminished or Normal. The finding of hypersensitivity will be collapsed into the category of "normal".

4) The Babinski sign is also known as a positive response to the plantar reflex test, which is used to assess motor neuron function. To test the reflex, the lateral, or outside, sole of the foot is stroked with a thumbnail or blunt instrument. A positive Babinski sign is when the big toe extends and the other toes fan out in an "upgoing" manner, which is considered an abnormal neurological response indicative of upper motor neuron damage.

5) Manual Muscle testing will be performed by having the patient contract the muscle against resistance of the clinician's hands. The standard clinical grading system will be used to rate the degree of muscle strength from a minimum of 0 (complete paralysis) to 5 (normal strength). We will test plantar flexion of the foot and dorsiflexion of the great toe and foot.

4. **Neurodynamic Exam**: this procedure is designed to place tension on the lumbar nerve roots by keeping one end of the neural tract stationary and applying tension to the other end. This is typically performed in seated position with the patient's neck and upper back in a flexed posture, while the clinician passively extends the knee and raises the lower extremity into hip flexion. Additional neural tension can be added by passively dorsiflexing the foot/ankle. A positive test is reproduction of significant leg pain/symptoms, which will be recorded as 2 possible categories of response: Positive, Negative. 2 nerves will be tested: Sciatic and Femoral. A positive test is defined as any reproduction of symptoms verbalized by the patient as a "familiar pain" in the leg or buttock region during performance of the test. The report of a feeling of "muscle tightness" will be recorded as a negative test.

5. **Hip and Knee exam**: We will apply the American College of Rheumatology (ACR) criteria for hip osteoarthritis (OA) and knee OA, recording whether or not the patient meets either/both of these criteria.

Hip Exam:

The patient will be lying in the supine position and the therapist will use a goniometer or inclinometer to measure (in degrees) the amount of internal rotation and flexion range of motion in each hip. The therapist will manual input the answer Yes or No on the iPad, for each ROM and each hip; using these criteria:

- Internal rotation: YES = < 15 degrees NO = > 15 degrees
- Flexion: YES = < 115 degrees NO = > 115 degrees

We will apply the American College of Rheumatology (ACR) criteria for hip osteoarthritis (OA) as defined below:

History of hip/groin pain for most days of the prior month, in addition to at least 3 of the following:

1. Pain present on internal hip rotation
2. Morning stiffness <60 minutes duration
3. Age > 50 years
4. Internal hip rotation < 15 degrees
5. Hip flexion < 115 degrees

The computer will automatically combine these physical exam findings with the previous self-report questions, with a pop-up window alerting the clinician whether these ACR criteria have been met for a diagnosis of hip OA.

Knee Exam:

The patient will be lying in the supine position and the therapist will proceed to palpate the medial and lateral joint lines of the knee with one hand, while passively flexing and extending the knee with the other hand. A "yes" response to crepitus is recorded if there is any audible or palpable clicking, grinding, or cracking during this procedure. The therapist will also record (yes or no) whether the palpation revealed any bony enlargement, tenderness, or warmth over the knee structures. We will apply the American College of Rheumatology (ACR) criteria for knee osteoarthritis (OA) as defined below:

Articular knee pain present for most days of the prior month, in addition to at least 3 of the following:

1. Crepitus on active joint motion
2. Morning stiffness <30 minutes duration
3. Age >50 years
4. Bony enlargement of the knee on examination
5. Bony tenderness of the knee on examination
6. No palpable warmth

The computer will automatically combine these physical exam findings with the previous self-report questions, with a pop-up window alerting the clinician whether these ACR criteria have been met for a diagnosis of hip OA.

6. **Lumbar Spine Exam**: this will involve the simple task of having the patient perform single movements of lumbar Active Range of Motion (AROM) in flexion and extension, observing the movements and asking the patient about pain provocation and peripheralization:

1. Was back pain provoked during extension?
2. Was back pain provoked during flexion?
3. Did you feel pain in your leg, calf or foot during extension?
4. Did you feel pain in your leg, calf or foot during flexion?

7. **Extended Short Physical Performance Battery (SPPB)**. We will administer an extended version of the SPPB. The published SPPB protocol is a reliable and valid set of tests that assesses 3 lower extremity performance subscales. We will add the 4th test: 1) Static standing balance; 2) Four meter gait speed; 3) Five repeated chair stands; 4) Ability to stand on one leg.

8. **Self-Paced Walking Test (SPWT)**. This is one of our primary outcome measures. Subjects will be instructed to walk continuously at their own pace until they feel they have to stop due to symptoms of LSS (or other reasons), or until the time limit of 30 minutes has been reached. Subjects will be asked to indicate when they first experience symptoms. When this happens they will be asked to indicate the nature of the symptoms. If symptoms are present at the onset of the test, they will be asked to indicate when they experience a significant increase in symptoms. Test termination will be defined as a complete stop of 3 seconds or more. A research assistant will walk with the subject, record this information about symptoms, and carry a portable chair upon which the subject may sit at

any time that the symptoms become too intense to continue walking. Data to be recorded will include: time/distance to onset of symptoms, nature of symptoms, total walking time, total distance walked, average walking speed, and the reason for test termination should they not walk for the full 30 minutes. If the subject is positive according with ACR criteria for hip and/or knee (from the findings of Clinical Symptoms and Knee and Hip Exam), then during the walk test the subject is queried for the location of the pain that caused them to stop walking. If the description of pain is congruent with hip/groin pain or knee joint pain - and was "that particular pain" - that forced them to stop walking, s/he is excluded from participation in the study.

Participants who meet the eligibility criteria following this baseline appointment will be set up with the SenseWear Armband to wear at home for one week.

9. **The SenseWear Armband** - Real-time physical activity will be measured by the SenseWear Professional v 6.1 (Body Media Inc, Pittsburgh PA). The SenseWear armband (SWA) is a small device that collects information from multiple sensors: a triaxial accelerometer, heat flux, skin temperature, and galvanic signal. The information is integrated and processed by software using proprietary algorithms utilizing subjects' demographic characteristics (gender, age, height, and weight) to provide minute-by-minute estimates of physical activity. The SWA has shown good reliability and validity. Subjects will be instructed on the proper application of the SWA on the upper arm at each assessment visit and given a unit to take home. Subjects will be instructed to wear the SWA on the back of the right arm during 24 hours/7 days (except during shower or water activities) to obtain 5 whole days of data. After 7 days of use, subjects will mail the device back in a pre-paid Priority Mail package.

Self-Reported Measures:

The research assistant provides the instructions and necessary assistance to subjects to complete the self-reported forms.

1. **Swiss Spinal Stenosis Questionnaire (SSS)**: This is our primary outcome measure. The SSS instrument is a patient self-report measure of pain and physical function. This instrument will be repeated at mid-treatment, post treatment, and long term follow up.

2. **Falls History**: patient self-report of falls over the past year (4-9 items).

3. **Modified Co-Morbidity Disease Index (MCDI)**: subjects will be asked to indicate any medical conditions with which they have been formally diagnosed by a physician in the past.

4. **Global Walking and Mobility Items**: self-rating of walking ability and mobility.

5**. Activities Specific Balance Confidence (ABC) Scale**: subject's level of self-confidence about performing certain activities of daily living without losing balance or becoming unsteady.

6. **Oswestry Low Back Pain Disability Index**: self-report measure of interference due to back pain.

7. **The RAND 36-Item Health Survey**: a validated and well-used measure of general Quality of Life.

8. **Tampa Scale for Kinesiophobia (TSK)**: self-report measure for assessing fear of movement and injury in back pain patients.

9. **Depression (PROMIS)**: 8-item short form PROMIS depression scale. Participants who have severe depression will be referred to the crisis center re:solve Network within Allegheny County, or other crisis centers outside of Allegheny County.

10. **Credibility/Expectancy Questionnaire (CEQ)**: patient's perceived credibility about the treatment and how much expectation there is that the treatment will be helpful.

6. RANDOMIZATION

The randomization will be performed *after* the baseline assessment. The study coordinator will carry out the randomization through web-based computer system used in this study. Randomization scheme is based on a minimization algorithm proposed by Stigsby and Taves[i]. The approach is to balance on multiple baseline prognostic factors using a rank-based method. Three variables will be used: baseline Swiss Spinal Stenosis Score (SSS), baseline Self-Paced Walk Test (SPWT), and age.

7. MASKING

While the treatment assignments obviously cannot be masked to the patient, all clinician-assessed evaluations post-intervention will be carried out by testers blinded to the patient's group assignment. Many of the outcome variables assessed in this study are patient self-report measures. One advantage of self-report measures is the minimization of tester bias. The performance-based variables in this study include the SPWT and the SPPB. Both self-report and performance-based variables will be assessed by testers who are not involved in the patient's treatment and are blinded to the treatment assignment. Since randomization will be performed after baseline assessment, the variables of physical exam performed at baseline to be explored as potential predictors of outcome should not be influenced by tester bias.

8. STUDY INTERVENTIONS

8.1. Usual Medical Care

Personnel performing the procedures: Dr. Ronald Glick, MD and/or Research Coordinator.
Location of the procedures: UPMC Center for Integrative Medicine, UPMC Shadyside.
Duration of the procedures: Three 30 minute examination visits over a period of approximately 6 weeks.

Procedures:
Subjects will see a board certified physical medicine and rehabilitation physician for a history and examination, after which a determination is made about a course of treatment that is individualized to the needs of each patient. The physician is permitted to prescribe any of the following oral medications, based upon the individual clinical presentation of each subject:

- Non-steroidal anti-inflammatory: ibuprofen, celocoxib, or diclofenac/misoprostol
- Adjunctive analgesics: acetaminophen, or gabapentin
- Antidepressant agents: nortriptyline, duloxetine, sertraline, trazodone, or mirtazapine

The physician also has the option to refer subjects for epidural steroid injections, which can be performed at a number of locations in Pittsburgh, including UPMC hospitals or out-patient centers. If recommended, epidurals are standard of care procedures and will be paid for by the study through an Institutional Account. In addition to oral medications and/or epidural injections, all subjects are given advice to stay active and a basic posture advice.

Subjects are asked about treatment compliance, side effects and adverse events at each visit, and complete the Swiss Spinal Stenosis Questionnaire and Oswestry Disability Index at mid-treatment (visit 2).

8.2. Community-based Group Exercise

Personnel performing the procedures: Senior physical fitness instructors at the participating community centers.
Location of the procedures: Jewish Community Center (JCC) in Squirrel Hill or South Hills or the Vintage Center in East Liberty, both local community senior centers which cater to the needs of older adults.
Duration of the procedures: Approximately 2 exercise classes per week (45 minutes each) for 6 weeks, for a total of 12 classes.

Procedures:
Subjects will attend group exercise classes designed specifically for older adults and taught by senior physical fitness instructors. Participants can make a choice between a class that provides chairs for seated or standing support or another class for people who are comfortable standing without any support. Each class consists of a variety of exercises designed to increase muscular strength, range of movement, and activity for daily living skills. There is a minimal amount of low impact cardiovascular exercise combined with the use of small hand-held weights, elastic tubing and gym balls for gentle resistance exercise.

The participants in these classes are monitored carefully for any signs of physical discomfort and are told to go at their own pace, resting whenever necessary. No specific body region is targeted with these exercise classes. The goal is to provide participants with gentle strength, balance, and generalized fitness training for the entire body. Some of the specific exercises included in these classes are: ankle/wrist rotations, partial squats, leg and knee extension/flexion, elastic tubing for strength training of the upper arm and chest muscles, coordination drills with a gym ball such as bouncing, throwing and catching.

Subjects are asked about treatment compliance, side effects and adverse events, and complete the Swiss Spinal Stenosis Questionnaire and Oswestry Disability Index at mid-treatment (at approximately 3 weeks).

8.3. Clinic-Based Manual Therapy and Rehabilitative Exercise

Personnel performing the procedures: Licensed physical therapists and/or chiropractors who have at least 5 years of clinical experience with manual therapy and rehabilitative exercise procedures.
Location of the procedures: Physical Therapy Clinical and Translational Research Center (PT-CTRC) which is an outpatient facility located on the campus of the University of Pittsburgh.
Duration of the procedures: Approximately 2 treatment sessions per week (45 minutes each) for 6 weeks, for a total of 12 visits.

Procedures:
This group of subjects are treated with a combination of manual therapy and rehabilitative exercise procedures that are well established in the physical therapy and chiropractic literature. A detailed written treatment protocol and clinical decision tree will be reviewed with each clinician, with the chiropractic co-investigator and consultant providing training sessions before recruitment to ensure treatment consistency. Treatment sessions include a pragmatic use of the individual methods listed below:

- Warm-up on a stationary exercise bicycle; time is individualized to tolerance of each subject
- Distraction-manipulation: this is a form of manually-assisted segmental lumbar traction manipulation using a specialized treatment table.
- Neural mobilization: this performed with the patient supine, applying repeated ankle dorsiflexion with the leg extended to generate tension-mobilization of the lumbosacral nerve roots.
- Hip, sacroiliac and lumbar facet mobilizations: these are standard manual mobilizations applied to improve segmental mobility in these joints.
- Soft tissue mobilizations of lumbopelvic and lower extremity muscles: these include deep pressure over taut bands/myofascial trigger points and post-isometric (contract-relax) stretching techniques.
- Self-stretching: patients are taught how to perform neural mobilization and self-stretches for home use
- Spinal stabilization exercise: these include exercises for core stability and spinal range of motion

Subjects are asked about treatment compliance, side effects and adverse events at each visit, and complete the Swiss Spinal Stenosis Questionnaire and Oswestry Disability Index at mid-treatment (approximately 3 weeks).

9. FOLLOW-UP VISITS

All participants will be asked to return to the PT-CTRC for two follow-up evaluations at approximately 2 weeks (Follow-Up 2 months) and 4 months (Follow-Up 6 months) following the 6-week treatment phase. Primary and secondary outcome measures and questionnaire data will be repeated. Each visit will take approximately one hour.

Questionnaires include self report measures of pain and physical function, and other psychosocial measures:

1. Swiss Spinal Stenosis Questionnaire (SSS)
2. Activities Specific Balance Confidence (ABC) Scale
3. Falls History
4. Oswestry Low Back Pain Disability Index
5. The RAND 36-Item Health Survey
6. Global Walking and Mobility Items
7. Patient Global Index of Change (PGIC) and Patient Satisfaction assess patients level of improvement and satisfaction with the treatment

Physical Exams will be performed by licensed Physical Therapists or Doctors of Chiropractic, and will include the following:

1. Self-Paced Walking Test (SPWT)
2. Short Physical Performance Battery (SPPB)
3. SenseWear Armband

10. ADVERSE EVENTS

10.1. Management

The occurrence of adverse events is monitored for each subject on an ongoing basis throughout the study. Reporting of adverse events in the context of the proposed program of research occurs according to the following definitions:

- Serious. This adverse event is fatal or life-threatening; requires hospitalization or produces a disability.
- Moderate or greater severity. This adverse event requires medical evaluation and/or medical treatment; or is a serious adverse reaction.
- Unexpected. This event is not identified in nature, severity or frequency in the IRB-approved research protocol or informed consent document.
- Associated with the research intervention. There is a reasonable possibility that this event may have been caused by the research intervention (i.e., a causal relationship between the event and research intervention cannot be ruled out by the investigators).

10.2. Report

All adverse events that are (a) unexpected; (b) of moderate or greater severity; and (c) associated with the research intervention are reported to the IRB. In the case of a serious adverse event, an emergency meeting of the investigative team is called. At the time of this meeting, a determination is made as to whether the trial should be prematurely interrupted. Expected adverse events; unexpected adverse events of minor severity; or adverse events which are determined by the PI to be unrelated to the research intervention are not reported to the IRB. These events are reported to PCORI during the annual report.

All adverse events are reported according to the following timeline: If the event is fatal or life-threatening, the report to the IRB and the PCORI occurs within 24 hours of the event. If the event is unexpected, and of moderate or greater severity (but not fatal or life-threatening), and associated with the research intervention, it is reported to the IRB and the PCORI within 10 calendar days of the reaction. The IRB and the PCORI are also notified as soon as possible of major disputes between the PI and/or project staff and a research subject or between research investigators (including research staff) involved in the proposed program of research if the resolution of the dispute is or will be problematic. If an unexpected adverse event occurs, the PIs re-assess the risk/benefit ratio of the study and submit any modifications deemed necessary to the IRB and the PCORI for approval.

11. STUDY COMPENSATION

Subjects are compensated for their time during treatment (up to $120) and at both follow up visits ($60 each).

12. SAMPLE SIZE JUSTIFICATION

Power analysis**:** A sample size of 150 subjects (n=50 per group) equates to over 80% power to detect a difference of 3.6 points on the SSS score (with a SD of 6.1 based upon preliminary data). In addition to these tests, the given sample size also yields sufficient power to fit a regression model that detects a statistical difference in the proportion of variability explained. More specifically, a sample size of 150 achieves 81% power to detect an R-square of 5% attributed to 2 independent variables (representing treatment) and assuming the control variables account for an additional 20% of the variability. We will recruit an additional 30 subjects to account for an anticipated drop-out rate of 15%.

13. DATA ANALYSIS

13.1. Descriptive Statistics

All primary analyses will be preceded by a descriptive analysis of baseline and outcome measures. Summary statistics will be presented both for the overall study cohort and stratified by treatment group. Although we expect that the randomization procedure will balance the treatment groups for all predictors, the significance of any imbalance will be assessed either using analysis of variance (for continuous measures) or chi-square test (for categorical measures). Descriptive statistics and visual summaries will be used to assess assumptions such a normality and linear association between outcomes and predictors of interest. If assumptions are not met, data transformations or more robust statistical procedures will be used.

13.2. Analytic Statistics

We will perform a number of statistical analyses of data obtained from the following 3 comparison groups in this trial:

- Group 1: Usual medical care
- Group 2: Community-based group exercise classes
- Group 3: Clinic-based manual therapy and rehabilitative exercise

Primary Aim: LSS subjects in Groups 2 and 3 will demonstrate greater improvement in self-reported pain/function and walking performance as compared to Group 1.

Statistical analysis 1**:** The primary outcome variables for this hypothesis are Swiss Spinal Stenosis (SSS) score and Self-Paced Walk Test (SPWT) measured post-intervention at 2 months, and both will be analyzed similarly. We will also assess these outcomes at 6 months. The outcome and all baseline characteristics will be summarized separately by treatment group. Analysis of Variance (ANOVA) will be used to assess the unadjusted association between the outcome variable and treatment groups. Multivariable linear mixed models will be used to assess the significance of treatment for each group while adjusting for the 3 randomization baseline variables. The linear

mixed models will include baseline data from all participants who were randomized, regardless of missing data after randomization, and therefore will follow an intention-to-treat approach.

Statistical analysis 2**:** We will also perform a responder analysis using dichotomous outcomes, consistent with the recommendations published by the Initiative on Methods, Measurement, and Pain Assessment in Clinical Trials (IMMPACT). Our responder analysis will compare the percentages of subjects who achieve meaningful outcomes between treatment groups on SSS score and SPWT (distance walked). Per IMMPACT recommendations, subjects who achieve at least 30% and 50% decreases in outcome measures are considered to show "moderate improvement" and "substantial improvement", respectively. Differences in the proportion of responders versus non-responders between the groups will be assessed using Chi-square tests.

Secondary Aim: Subjects in Groups 2 and 3 will demonstrate a greater change in physical activity compared to Group 1.

Statistical analysis 1**:** The primary outcome variable for this hypothesis is physical activity data captured by the SenseWear Armband measured post-intervention at 2 months. To compare changes in physical activity levels between the three treatment groups linear regression analysis will be used. Univariate regression models will be used to assess the unadjusted association between the outcome variable and treatment groups. Multivariable linear mixed models will be used to assess the significance of treatment for each group while adjusting for the 3 randomization baseline variables. The linear mixed models will include baseline data from all participants who were randomized, regardless of missing data after randomization, and therefore will follow an intention-to-treat approach.

Statistical analysis 2**:** We will also perform a responder analysis using dichotomous outcomes, consistent with the recommendations published by the Initiative on Methods, Measurement, and Pain Assessment in Clinical Trials (IMMPACT). Our responder analysis will compare the percentages of subjects who achieve meaningful outcomes between treatment groups on the changes in their average amount of daily physical activity (SWA). Per IMMPACT recommendations, subjects who achieve at least 30% and 50% increase in their level of mean daily physical activity are considered to show "moderate improvement" and "substantial improvement", respectively. Differences in the proportion of responders versus non-responders between the groups will be assessed using Chi-square tests.

Exploratory Aim 1: For all 3 treatment groups there will be a low incidence of adverse events and similar treatment adherence rates. The 6-month attrition rate, number of falls, will be lower in Groups 2 and 3 compared to Group 1.

Statistical analysis**:** Descriptive analysis of 6-month attrition rates, number of adverse events, treatment adherence rates, number of falls, will be conducted using simple frequencies and contingency tables for associations with treatment group. Although these analyses will focus on descriptive summaries and judgments of clinical important differences, chi-squared or Fisher's exact tests will also be conducted to quantify the corresponding significance levels.

Exploratory Aim 2: Heterogeneity of treatment effect. A group of baseline physical, psychosocial, and demographic measures will be associated with treatment response and non-response in each group. We expect baseline predictors of treatment response to be different in Group 1 as compared to Groups 2 and 3.

Statistical analysis: Our second exploratory aim was to explore potential baseline predictors of treatment response. Univariate analyses were first performed to test for possible associations between baseline characteristics and response, based upon the criterion of responders being defined as those participants who demonstrated ≥30% change on either the SSS or SPWT. Two-sample t-tests for continuous variables and chi-squared tests for categorical variables were used to test the association between responders/non-responders and each variable of interest. We decided a priori that statistical significance would be set at p <0.10 for variables to be included in the multivariable logistic regression model. We used the same responder/non-responder dichotomous variables for SSS, SPWT and physical activity created for the secondary responder analyses associated with our primary and secondary aims.

Logistic regression models were then used to test the association between responders/non-responders, treatment groups and other variables of interest. The variables that were found significant using univariate procedures (p<0.10) were put in a multivariable logistic regression model and a backward elimination procedure was applied. The level of significance for removal was p < 0.10.

We evaluated heterogeneity of treatment effects by testing the interaction between treatment and 8 baseline variables using multiple logistic regression models with responder status as the dependent variable. These 8 variables were: age, sex, BMI, comorbidities, race, kinesiophobia, knee osteoarthritis and depression. Age was dichotomized at <75 years versus ≥75 years. Comorbidities, depression and the other continuous variables were split at their respective medians. The baseline variables were considered as potential moderators of treatment effect due to our experience in other studies and the published literature. The p-value for the interaction between the moderator and the treatment variable was compared

Regardless of significance, between treatment groups comparisons were presented as odds ratios and 95% confidence intervals stratified by the potential moderator.

MISSING DATA

To account for any missing data, we will use linear mixed effects models to study treatment differences over time for the primary and secondary outcomes. This methodology employs a compound symmetry structure for the correlation structure and the Kenward-Roger approximation method for the degrees of freedom[57]. Linear mixed effects models use all available data; if a subject has a measurement at one time-point and the rest of the data is missing, that subject was still used in the analysis. Therefore, these mixed models included data from all participants who had a baseline examination and were randomized (N=259), and follow the intention-to-treat principle.

14. RECORD KEEPING

The majority of the forms and questionnaires used in this study are entered directly into the database. The hard copy of the forms completed on paper are being stored in subject's file and kept in a locked file cabinet in the trial coordinator's office. Only study personnel has access to these files.

A copy of the Informed Consent signed by the subject and by the trial coordinator is kept in a separate locked file cabinet in the trial coordinator's office, as well as the Contact Information form, Telephone screening form, and any medical record data provided by the subject containing identifiable information.

The database is web-based for direct data entry, where subjects are identified by a study ID, and no personally identifiable information is stored in it or used in any of the analyses.

15. DATA MANAGEMENT PLAN

Data management is overseen by the PI, Research Coordinator, and is coordinated by the Office of Academic Computing (OAC), University of Pittsburgh. The OAC created an electronic System for Data Management WebDataXpress (WDX)) for data collection, tracking, follow-up, reporting, and analysis. The WDX system includes verifying the data, out of range data checks, and repeated evaluation of data process, eliminating the possibility of most incorrect entries and preventing extensive recoding and cleaning by the statistician.

The primary method of data collection is through the database. However, if access to the internet is disrupted, paper forms are available to ensure data collection. All data collected in paper forms are stored in the subject's research chart identified by their ID. In this case, coordinator contacts the database programmers via telephone to obtain the subject's group assignment.

16. QUALITY ASSURANCE

To ensure data quality and integrity we are using standard methods of data collection and recording, have formal staff workshops on research integrity, document computer operations and data editing procedures, and have regular meetings with project staff to review any changes in procedure. The electronic forms are maintained by OAC on a local network in a relational database. All files are backed-up daily and archived weekly.

17. HUMAN SUBJECTS

17.1. Institutional Review Board (IRB) Review and Inform Consent

This protocol and the informed consent document and any subsequent modifications will be reviewed and approved by the University of Pittsburgh IRB (PRO12120422). A signed informed consent form is obtained from all subjects. The consent form describes the purpose of the study, the procedures to be followed, and the risks and benefits of participation. Subjects will be informed that they are free to withdraw from the study at any time. A copy of the signed informed consent form is given to the subject.

17.2. Potential Risks/Risk Management

<u>Risks associated with Interviews and Questionnaires:</u> Completing the questionnaires may cause subjects to feel uncomfortable when answering questions of a personal nature. To minimize this risk, subjects may choose not to answer any of the questions regardless of reason.

<u>Risks associated with baseline physical examination</u>: The risks associated with the physical examination procedures may include temporary muscle soreness or tripping and falling during testing and/or exercises.

During testing and training, risks of tripping and falling will be minimized by providing direct stand-by supervision by the research staff. In addition, the exclusion criteria used in this study provides that individuals who are prone to falling, or have a neurodegenerative disorder will not be participating in the study. When engaging in exercise, there is a rare risk that subjects may experience chest pain, dizziness, shortness of breath, heart attack, or stroke.

Risks associated with manual therapy and exercise: The most common side effects of manual therapy and exercise include mild discomfort and soreness in the lower back, hip, or buttock regions that usually goes away within a day or two. More serious complications are extremely rare and their association with spinal manipulation is debated. These complications include nerve damage, injuries to spinal discs, and spinal fractures. The chance of serious complications is estimated to be 1 per million manipulations of the lower back. When engaging in exercise, there is a rare risk that subjects may experience chest pain, dizziness, shortness of breath, heart attack, or stroke. To minimize this risk, participants are instructed not to exert themselves beyond their level of physical comfort and that they should inform the exercise instructor or clinician about any of these symptoms.

Risks associated with usual medical care: The most common risks are potential side effects to various medications. All subjects in this treatment arm will be examined and monitored by a physician who is board certified in physical medicine and rehabilitation, and psychiatry. He will conduct a thorough case history and carefully ask patients about their current medications, as well as any side effects or allergies they've had with previous medications. He will also monitor all patients for any medication side effects and discontinue or change any medications that are not well tolerated.

Pain medications may include tramadol or gabapentin. There are relatively little side effects associated with the normal use of Tylenol; the most serious and rare side effect of Tylenol use is liver damage due to large doses, chronic use, or taking Tylenol along with large amounts of alcohol or other drugs that may damage the liver. Common side effects of tramadol include: Constipation, dizziness, headache, nausea, vomiting, decreased lung function. Common side effects of gabapentin include: Drowsiness, dizziness, low energy, muscle pain, fluid retention, blurred vision.

Anti-inflammatory medications may include any of the following: ibuprofen, naproxen, or celecoxib. Common side effects of these medications include: Tinnitus, rash, hives, water retention, drowsiness, dizziness, headache, nausea, heart burn, stomach cramps, swelling.

Mood/sleep medications may include any of the following: Nortriptylin, Trazodone, Duloxetine or Venlafaxine. Common side effects of these medications are: dry mouth, low blood pressure, constipation, drowsiness, dizziness, low energy, nausea, vomiting, headache, anxiety, sexual problems, loss of appetite, insomnia, diarrhea.

If the doctor recommends an epidural injection, there are other potential side effects and risks, most of which are considered rare. Post-injection infections from the needle puncture can occur, but are very rare. Post-injection bleeding is another rare risk, which is more common in patients taking blood thinner medications. Direct damage to a spinal nerve from the needle is another rare risk, and associated only with one type of nerve block procedure. Temporary reduction of bladder tone from the anesthetic effect on the nerves is also possible.

In rare cases there may be a leakage of spinal fluid from the needle puncture associated with post-injection headache, requiring a blood patch to stop the leakage.

17.3. Privacy

All baseline and follow-up research activities will take place in a private room at the Physical Therapy Clinical and Translational Research Center. The data collected will be limited to information necessary to achieve the aims of this research. PT treatment sessions take place in an open treatment room with the necessary equipment due to the nature of the treatment. Participants are not specifically identified as part of this research study; but note that these treatment sessions take place in the PT-CTRC and all individuals receiving therapy in this location are participating in some type of research protocol.

Participants in the Usual Medical Care group will be seen at the UPMC Center for Integrative Medicine in a private room. They will not be identified as research participants in the reception room or waiting areas. Participants in the group exercise intervention will be provided with a membership to the JCC or Vintage Center and will be treated in the same manner as other regular members of the facility. They will not be identified in their exercise classes in any way as research participants. The administrative teams at the JCC and Vintage Center have completed the approved CTSI Community Partner Research Ethics training program with the PI.

17.4. Confidentiality

Patient confidentiality is maintained throughout the study. The risk of breaching subject confidentiality is minimized by using a web-based system of data entry. The data is directly entered into a computer at the time of the interviews. A relational database is stored on a local network where only select research team members have access. All study subjects are assigned unique study identifiers that appear on all data collection instruments, documents, and files used in the statistical analysis and manuscript preparation. Any items containing identifiable information, such as patient names on consent forms, will be stored in a locked file cabinet in a separate location from data forms. Only limited team members have access to personal information needed for tracking and informed consent. No personal information concerning study participants will be released without their written consent.

17.5. Potential Benefits of the Proposed Research to Human Subjects and Others

All of the treatments provided in the three arms of this study have the potential to provide direct benefit to the research participants. The oral medications and spinal injections are commonly used by physical medicine and rehabilitation physicians to treat patients with lumbar spinal stenosis. Community based group exercise classes - such as the popular Silver Sneakers program - have been taught to older adults around the country for many years with good success. Although not specifically designed for patients with lumbar spinal stenosis, these group exercise classes have the potential of improving physical fitness, balance and walking performance in those who participate. Clinic based physical therapy and chiropractic treatment have been shown in several observational studies to benefit some patients with lumbar spinal stenosis.

17.6. Importance of the Knowledge to be Gained

Many patients with LSS are poor candidates for spine surgery due to high risk from co-morbidities such as diabetes, obesity and cardiovascular disease. In addition, a large number of LSS patients with mild or moderate symptoms and functional limitation simply do not require surgical intervention. At this point, little is known about their

chances of improving with non-surgical treatment. Patients who cannot - or choose not - to have surgery need more information to help make informed decisions about the effectiveness of the various non-surgical treatment options available to them for managing their LSS symptoms.

This is the basis for the Primary Aim of our study: to compare the clinical effectiveness of three pragmatic non-surgical treatment approaches.

APPENDIX *(documents from appendix are available upon request)*

A. Study Forms
B. Informed Consent
C. Study Advertisement
