# Supplementary material for: Comparative Clinical Effectiveness of Nonsurgical Treatment Methods in Patients With Lumbar Spinal Stenosis: A Randomized Clinical Trial
Source: JAMA Netw Open. 2019 Jan 4;2(1):e186828. doi: 10.1001/jamanetworkopen.2018.6828 (PMC6324321; doi:10.1001/jamanetworkopen.2018.6828)
Supplement: Supplement 2. — eAppendix. Trial baseline adaptive randomization methodology [file jamanetwopen-2-e186828-s002.pdf]

Supplementary Online Content

Schneider MJ, Ammendolia C, Murphy DR, et al. Comparative clinical effectiveness of nonsurgical treatment methods in patients with lumbar spinal stenosis: a randomized clinical trial. *JAMA Netw Open*. 2019;2(1):e186828. doi:10.1001/jamanetworkopen.2018.6828

eAppendix. Trial baseline adaptive randomization methodology

This supplementary material has been provided by the authors to give readers additional information about their work.

© 2019 Schneider MJ et al. *JAMA Network Open.*

eAppendix. Trial Baseline Adaptive Randomization Methodology

The method of allocation was a baseline adaptive randomization using a combination of rank minimization for balanced assignment (Stigsby and Taves, 2010) and minimization with probabilistic assignment (Pocock and Simon, 1975). The Stigsby and Taves (2010) approach is deterministic with zero tolerance for imbalance such that the probability of assignment to the allocation creating the least imbalance in the baseline covariates is 1. Following Pocock and Simon (1975), we modified the rank minimization method by adding an element of randomization where the probability of assignment varied depending on the number of groups with imbalance (biased coin approach). We selected this method due to concerns about selection bias due to the predictability of the assignments. We based the allocation on 3 continuous variables measured at baseline: SSS, SPWT, and age. The first 3 assignments were randomly assigned 1:1:1.

The following steps are repeated for each newly enrolled participant after the first 3 are assigned:

1. **Ranking of Participants Based on SSS, SPWT and Age**

At any point in the study after the first 3 participants are randomized, consider the currently enrolled participants (n) with measurements on the 3 baseline variables of SSS, SPWT and age. These *n* participants have already been assigned to an intervention group. A new participant is enrolled and these 3 baseline variables are measured. The participants are then ranked for each of these 3 baseline variables such that each participant has a ranking for SSS, SPWT, and age that ranges from 1 to n+1.

2. **Imbalance Score**

Each new participant is tentatively assigned to one of the three intervention groups. For each tentative assignment, the rank-sums for each variable are calculated per treatment group and these rank-sums are averaged to calculate the mean rank-sum. For our study, there were three baseline variables and three groups so 9 rank-sums would be calculated for each tentative assignment. The imbalance score for the tentative assignment is the sum of the squared deviations of the rank-sums for each variable per treatment group from their corresponding mean rank-sum. The imbalance scores are compared across tentative assignments and assignment for the new participant is made probabilistically.

3. **Probabilistic Assignment**

Following Pocock and Simon (1975), we modified the rank minimization method by adding an element of randomization where the probability of assignment depended on the number of tentative assignments resulting in minimal imbalance. The probabilistic

© 2019 Schneider MJ et al. *JAMA Network Open.*

approach ensured the next assignment could not be predicted with certainty. If there was no imbalance, the probability of assignment was uniform; if 1 group resulted in the least imbalance, the probability of assignment was 2/3 and 1/6 for each of the other two groups; if 2 groups tied for the least imbalance, the probability of assignment was 44% in each of the 2 groups and 12% in the remaining group.

The method used for our trial successfully meets the recommendation of managing balance and randomness while preventing or minimizing the potential for selection bias (Zhao & Berger 2018). In addition, we have controlled for the allocation factors (SSS, SPWT, age) in the analyses (see Statistical Analysis section of our methods and Scott et. al. 2002).

Citations:

- Stigsby B, Taves DR. Rank-Minimization for balanced assignment of subjects in clinical trials. *Contemp Clin Trials*. 2010; 31(2):147-50.
- Pocock SJ, Simon R. Sequential treatment assignment with balancing for prognostic factors in the controlled clinical trial. *Biometrics*. 1975; 31(1):103-115.
- Scott NW, McPherson GC, Ramsay CR, Campbell MK. The method of minimization for allocation to clinical trials. a review. *Control Clin Trial*s. 2002; 23(6):662-74.
- Zhao W, Berger V (2018) Imbalance control in clinical trial subject randomization from philosophy to strategy. *J Clin Epidemiol*. 2018; 101:116-118

© 2019 Schneider MJ et al. *JAMA Network Open.*
