# Supplementary material for: Comparative Clinical Effectiveness of Nonsurgical Treatment Methods in Patients With Lumbar Spinal Stenosis: A Randomized Clinical Trial
Source: JAMA Netw Open. 2019 Jan 4;2(1):e186828. doi: 10.1001/jamanetworkopen.2018.6828 (PMC6324321; doi:10.1001/jamanetworkopen.2018.6828)
Supplement: Supplement 3. — Data Sharing Statement [file jamanetwopen-2-e186828-s003.pdf]

Data Sharing Statement

Schneider. Comparative Clinical Effectiveness of Nonsurgical Treatment Methods in Patients With Lumbar Spinal Stenosis. *JAMA Netw Open*. Published January 04, 2019. 10.1001/jamanetworkopen.2018.6828

Data
Data available: Yes
Data types: Deidentified participant data, Other (please specify)
Additional Information: We are willing to share de-identified data with other researchers who have specific aims that would warrant a data use agreement.
How to access data: We will make the data available upon request and upon approval of a data use sharing agreement.
When available: With publication

Supporting Documents
Document types: Other (please specify)
Additional Information: We are willing to share any of the above supporting documents.
How to access documents: We will make these documents available upon request from other researchers.
When available: With publication

Additional Information
Who can access the data: We are willing to share de-identified data with any research who has a scientifically sound research question that would benefit from our data.
Types of analyses: Same as above. This includes any type of scientifically valid statistical analysis.
Mechanisms of data availability: We will make the data available after approval of a data use sharing agreement between the University of Pittsburgh office of research and the counterpart office at any other recognized university or research organization.
